# The cognitive compass of attachment: how primed security and insecurity navigate mental representations

**DOI:** 10.3389/fpsyg.2026.1713752

**Published:** 2026-02-06

**Authors:** Anna Kamza

**Affiliations:** Center for Research on Personality Development, Institute of Psychology, SWPS University, Warsaw, Poland

**Keywords:** attachment anxiety, attachment avoidance, attachment priming, attachment system, cognitive accessibility, individual differences

## Abstract

**Background:**

This study examined the domain-specific patterns associated with supraliminal attachment priming on the cognitive accessibility of attachment-related mental representations.

**Methods:**

Seventy participants were randomly assigned to one of three experimental conditions: attachment-insecurity priming, attachment-security priming, or a non-attachment control (non-primed reference) condition. Participants underwent supraliminal priming via a guided imagery task specific to their condition, followed by a lexical decision task measuring reaction times for five word categories: proximity-related, distance-related, positive, negative, and neutral words.

**Results:**

Relative differences between priming conditions emerged exclusively for attachment-related word categories. Participants in the attachment-insecurity priming condition showed faster reaction times to both proximity- and distance-related words relative to the non-primed reference condition. In contrast, participants in the attachment-security priming condition showed faster reaction times to proximity-related words than the non-primed reference condition only at low levels of attachment anxiety; no such differences were observed at higher levels of anxiety. Reaction times to distance-related words did not differ between the security priming and non-primed reference conditions. Attachment avoidance did not moderate any effects. Bayesian analyses provided affirmative evidence for the absence of priming effects in positive, negative, and neutral word categories. Given the observed effect sizes, moderation trend-level patterns should be interpreted as exploratory.

**Conclusion:**

These findings advance understanding of attachment system dynamics by showing that differences between insecurity and security priming in attachment-related processing depend on attachment anxiety.

## 1 Introduction

Attachment theory conceptualizes close relationships as regulated by an innate psychobiological attachment behavioral system that becomes activated under threat or distress and biases cognition toward proximity- and safety-related representations (Bowlby, 1969, 1988; Mikulincer and Shaver, 2007a, b). Through repeated interactions with caregivers, individuals develop internal working models (IWMs)—relational schemas that encode expectations about the self and others and guide emotion regulation and information processing in interpersonal contexts (Baldwin, 1992; Mikulincer and Shaver, 2007a). Importantly, IWMs guide emotion regulation and influence early cognitive processes, including attention, memory, and lexical access. Research has shown that attachment-related cues can bias the momentary accessibility of relational concepts, particularly those linked to proximity and distance (Baldwin et al., 1996; e.g., Mikulincer et al., 2000; Mikulincer and Shaver, 2020). Therefore, a central implication of this framework is that attachment-related cognitive structures can be experimentally activated.

However, most prior work has focused either on subliminal threat primes or on security priming examined in isolation, leaving open questions about how consciously processed (supraliminal) security and insecurity cues differentially affect lexical accessibility of attachment-related representations. The present study addresses this gap by examining how supraliminal attachment security, insecurity, and neutral priming influence reaction times to proximity- and distance-related words in a lexical decision task, and whether these effects are moderated by attachment anxiety and avoidance. Understanding these processes is important because proximity and distance are foundational dimensions of the attachment system and represent core relational tendencies linked to security and defensive regulation.

## 2 Literature review and hypothesis development

### 2.1 Dynamic functioning of attachment styles: integration of internal working models with attachment orientations

Attachment-related knowledge structures vary not only in their content but also in their momentary accessibility. Individuals typically possess multiple internal working models representing different relational experiences, including relationship-specific and more generalized schemas (Baldwin et al., 1996). While multiple representations may coexist in memory, only a subset is activated at any given moment, shaping ongoing cognition and behavior. Accessibility refers to this current activation level and determines the extent to which attachment-related expectations influence information processing (Mikulincer and Shaver, 2020).

Attachment orientations are associated with characteristic patterns of accessibility—the likelihood that specific relational expectations and scripts will be activated in social situations (Mikulincer and Shaver, 2003). Secure individuals hold positive models of self and others. They flexibly engage proximity-related cognition when support is needed and maintain balanced representations of closeness and autonomy (Mikulincer and Shaver, 2003, 2007a). However, attachment anxiety is linked to chronic hyperactivation of attachment system, and thus to heightened vigiliance to threat and hyperaccessibility of insecurity-related representations such as, e.g., rejaction-related content (Shaver and Mikulincer, 2002; Dewitte et al., 2008). These patterns may interfere with effective emotion regulation and amplify interpersonal distress (Mikulincer and Shaver, 2003).

On the other hand, attachment avoidance is associated with deactivating strategies that suppress attachment proximity-related content, particularly when consciously exposed to relational cues. However, this suppression may break down under cognitive load (Fraley and Waller, 1998; Mikulincer and Shaver, 2003; Fraley et al., 2013).

These regulatory tendencies suggest that attachment anxiety and avoidance may differentially shape responses to attachment priming, particularly when primes are consciously processed. From this perspective, situational cues can transiently alter the accessibility of attachment-related representations, providing a basis for experimental manipulation.

### 2.2 Attachment priming: experimental activation of working models

Attachment priming refers to experimental procedures designed to temporarily activate attachment-related representations, thereby increasing their momentary accessibility and enabling causal tests of attachment system functioning (Mikulincer and Shaver, 2007a; Mikulincer et al., 2011). By manipulating situational cues, priming paradigms allow researchers to examine how attachment-related knowledge structures shape cognition independently of stable attachment orientations.

Attachment priming studies have employed both subliminal and supraliminal procedures, which differ in the extent to which attachment-related cues are consciously processed. Subliminal primes are thought to activate attachment representations outside awareness and primarily engage automatic processes, whereas supraliminal primes allow for conscious elaboration and the involvement of regulatory strategies linked to attachment orientations (e.g., Mikulincer et al., 2000). This distinction is theoretically relevant for the present study, as it suggests that individual differences in attachment anxiety and avoidance are more likely to shape cognitive accessibility following supraliminal priming.

Consistent with prior work, insecurity priming reliably enhances the accessibility of attachment-related content, including proximity- and separation-related representations (Mikulincer et al., 2000; Shaver and Mikulincer, 2002). In contrast, the cognitive effects of security priming appear more conditional. Although security primes consistently increase felt security and support adaptive emotion regulation, their impact on early stages of cognitive processing is less uniform and may depend on dispositional attachment orientations (Gillath et al., 2008; Carnelley and Rowe, 2010; Rowe et al., 2020). In this line of research, cognitive accessibility has typically been operationalized using lexical decision tasks, in which faster reaction times to attachment-related words indicate heightened accessibility of the corresponding representations (Mikulincer et al., 2000).

To date, research has not directly compared security, insecurity, and neutral priming conditions within a single supraliminal paradigm using lexical measures of cognitive accessibility. Consequently, it remains unclear how consciously processed security and insecurity cues differentially influence the accessibility of core attachment-related representations, such as proximity and distance, and whether these effects are moderated by attachment anxiety and avoidance. Addressing these questions provides the rationale for the present study.

Accordingly, supraliminal attachment priming offers a theoretically informative context for examining how situational activation of the attachment system interacts with chronic attachment orientations, with reaction times to proximity- and distance-related words providing a sensitive index of these accessibility shifts.

### 2.3 Priming type and attachment orientations in the accessibility of proximity- and distance-related representations

Attachment-related cognitive accessibility is shaped by both situational activation of the attachment system and chronic attachment orientations. When attachment insecurity is activated, threat-related cues reliably increase the accessibility of attachment-relevant representations, reflecting a normative attachment response that involves heightened readiness for proximity seeking as well as sensitivity to relational separation (Mikulincer et al., 2000, 2002; Rowe et al., 2020; Gillath et al., 2025). Lexical decision and related paradigms have shown that insecurity-related primes facilitate access to proximity-related concepts and, in some cases, to separation- or distance-related representations, indicating concurrent activation of approach- and withdrawal-related tendencies within the attachment system (Clear and Zimmer-Gembeck, 2017; Sakman and Sümer, 2018).

In contrast, the cognitive consequences of security activation appear more selective. Although security priming is consistently associated with increased felt security and adaptive regulation, its effects on early stages of cognitive processing are less robust and may depend on individual differences in attachment orientation (Gillath et al., 2008; Carnelley and Rowe, 2010; Rowe et al., 2020). When security cues are processed consciously, their influence on cognitive accessibility may be shaped by attachment anxiety, which has been linked to heightened vigilance to relational cues and ambivalent responses to closeness (Shaver and Mikulincer, 2002; Long et al., 2020; Mikulincer and Shaver, 2020).

Distance-related representations reflect a distinct dimension of attachment-related processing associated with withdrawal, boundary regulation, and defensive strategies. Insecurity activation is expected to increase the accessibility of distance-related concepts as part of a broader threat response, whereas security priming is less likely to enhance such accessibility relative to a neutral baseline (Mikulincer et al., 2002; Clear and Zimmer-Gembeck, 2017). Attachment avoidance may further modulate responses to consciously processed attachment cues by promoting suppression or disengagement from attachment-related content, particularly in supraliminal paradigms (Fraley and Waller, 1998; Andriopoulos and Kafetsios, 2015; Mellor and Psouni, 2021).

Taken together, prior work suggests that attachment-related cognitive accessibility is jointly shaped by situational activation of the attachment system and chronic attachment orientations, with insecurity producing robust accessibility effects and security yielding more conditional outcomes. When attachment cues are processed supraliminally, these effects are expected to differ for proximity- and distance-related representations and to be moderated by attachment anxiety and avoidance. On this basis, the present study was designed to directly test these predictions using a lexical decision task.

Although attachment security priming has been shown to reliably increase subjective felt security and facilitate emotion regulation, evidence for its effects on early, automatic indices of cognitive accessibility is more mixed. Prior work suggests that security priming may operate primarily as a regulatory buffer, rather than as a direct enhancer of associative activation, particularly in the absence of contextual threat. Consequently, any facilitation of proximity-related representations at the level of early lexical access may be relatively subtle and contingent on individual differences in attachment-related regulation.

### 2.4 Present study

Attachment theory emphasizes the role of internal working models in social information processing, with attachment system activation affecting cognitive accessibility. In the context of attachment priming research, lexical decision reaction times provide an index of the momentary accessibility of attachment-related representations. Differences in response latencies to proximity- and distance-related words are therefore interpreted as reflecting shifts in the activation of core attachment schemas following situational cues, with proximity-related words reflecting secure base activation and distance-related words indexing withdrawal- and separation-related schemas.

However, the specific impact of distinct supraliminal priming conditions (security, insecurity, control) on the cognitive accessibility of fundamental attachment concepts requires further investigation. While studies have examined priming effects on attention and recall and compared security priming to control or threat conditions, a systematic comparison targeting proximity and distance accessibility (via LDT) remains incomplete. Although individual differences in attachment anxiety and avoidance influence information processing and moderate priming effects, the precise pattern of proximity and distance representations across explicit priming conditions requires clarification.

The present study aimed to address these gaps by comparing the effects of supraliminal attachment security- and insecurity priming, and a control condition on the cognitive accessibility of proximity and distance representations, as measured by LDT. A general attachment-insecurity prime was used instead of separate anxiety and avoidance primes to activate the attachment system without biasing participants toward a specific regulatory strategy. This approach reflects the shared core of insecure attachment—perceived unavailability or inconsistency of close others—and allows for broader generalizability (Mikulincer and Shaver, 2007a; Rowe et al., 2020). It is particularly suitable for supraliminal priming, where conscious processing may interact with individual attachment histories.

Accordingly, the hypotheses tested in the present study were derived from attachment theory’s distinction between normative insecurity-driven activation of attachment representations and more conditional, regulation-dependent effects of security activation under supraliminal processing (Mikulincer et al., 2000). Based on this theoretical framework, I hypothesized that priming conditions would differentially affect the accessibility of proximity- and distance-related words, with these effects further modulated by attachment anxiety and avoidance. Specifically, (H1a) both attachment insecurity and security priming were expected to increase proximity-related word accessibility compared to the control condition, as shown by faster LDT reaction times. From an attachment-system perspective, activation of insecurity constitutes a normative stress response (regardless of chronic attachment style) that reliably increases the accessibility of proximity-related representations, reflecting heightened readiness for support seeking (Mikulincer et al., 2000; Mikulincer and Shaver, 2003). In contrast, the effect of security priming on proximity-word accessibility is theoretically derived rather than empirically robust as it has received limited empirical support. As security-related representations are assumed to be embedded in associative networks linking safety, support, and closeness (Mikulincer et al., 2001), my hypothesis that security priming facilitates the accessibility of proximity-related words is primarily grounded in theoretical assumptions about the associative structure of attachment-related cognitive networks, rather than in consistent prior empirical findings.

Distance-related representations index a distinct dimension of attachment-related processing associated with withdrawal, boundary regulation, and perceived relational threat. Prior work suggests that insecurity activation simultaneously heightens sensitivity to both proximity seeking and separation-related cues, reflecting ambivalent approach–avoidance dynamics within the attachment system (Mikulincer et al., 2002; Clear and Zimmer-Gembeck, 2017). In contrast, security activation is not expected to directly enhance the accessibility of distance-related concepts relative to a control condition, as security primarily operates as a regulatory buffer rather than a trigger of withdrawal-related schemas. Thus, I predicted (H1b) that insecurity priming would increase distance-related word accessibility compared to control and security conditions, as it activates negative attachment representations leading to schema-congruent processing biases.

Given the limited evidence for automatic activation of security-related representations at early stages of lexical processing, it cannot be ruled out that their accessibility depends on regulatory context rather than occurring reflexively. Accordingly, any facilitation of proximity-related lexical access following supraliminal security priming was expected to be more conditional than the corresponding insecurity effect. Therefore, I hypothesized (H2a) that the facilitative effect of security priming on proximity concepts accessibility would be attenuated or absent for individuals with high attachment anxiety and avoidance. For anxious individuals, supraliminal security priming might lead to rumination or increased distance word accessibility, counteracting proximity word effects. Security priming’s benefits on emotional processing are weaker in highly anxious individuals during explicit priming (Mikulincer et al., 2011). For avoidant individuals, supraliminal priming is often less effective due to defensive mechanisms (Mikulincer et al., 2009), likely producing minimal effect on the accessibility of attachment-related words.

Furthermore, although insecurity activation is expected to robustly increase the accessibility of distance-related representations as part of a threat response, individual differences in attachment anxiety and avoidance may shape the qualitative expression of this effect under supraliminal conditions. Given the normative nature of insecurity-driven activation, moderation effects were expected to be secondary to the main effect of insecurity priming and therefore potentially weaker or context-dependent. In anxious individuals, heightened sensitivity to relational threat may further amplify separation-related accessibility, whereas in avoidant individuals, defensive disengagement may attenuate or complicate lexical access due to inhibition costs (Mikulincer et al., 2000, 2002, 2011). Hence, insecurity priming’s effect on distance word accessibility was hypothesized to interact with attachment orientations (H2b). In individuals with high anxiety, insecurity priming was expected to further increase distance word accessibility compared with individuals with low attachment anxiety. For individuals with high avoidance, supraliminal insecurity priming might trigger defensive processing (Mikulincer et al., 2002; Gillath et al., 2009), causing slower reaction times for attachment-related words due to inhibition costs. This would contrast with low-avoidance individuals’ straightforward increase in distance-related word accessibility.

Attachment priming is theorized to selectively activate attachment-related cognitive networks rather than producing generalized affective facilitation. Accordingly, (H3) I expected attachment priming and orientation effects to be specific to attachment-related representations (i.e., proximity, distance) that map directly onto attachment-system dynamics, while leaving processing of positive, negative, and neutral concepts unaffected (Mikulincer et al., 2000) Demonstrating such domain specificity is particularly important in supraliminal paradigms, where conscious emotional processing could otherwise produce diffuse affective effects.

The innovative aspect of this research lies in its comparative design, which simultaneously examines security priming, insecurity priming, and a control condition within a single experimental paradigm. While utilizing single-session rather than repeated priming, this approach eliminates between-study confounds, provides standardized conditions across priming types, and enables direct comparison of differential immediate effects. If the hypothesized relationships are confirmed, these findings could inform targeted therapeutic interventions while also establish a methodological foundation for future research.

## 3 Materials and methods

### 3.1 Participants

*A priori* power analysis was conducted using G*Power 3.1 (Faul et al., 2009) to determine the sample size necessary for the main analyses. For the MANOVA with three experimental conditions, five dependent variables (reaction times for different word types), and six predictors (including attachment dimensions, their interactions with condition and general trait-anxiety as control variable) and based on the literature (Rowe et al., 2020), I specified a medium-to-large effect size of *f^2^(V)* = 0.14 (Cohen, 1988; Huberty and Olejnik, 2006), an alpha level of.05, and a desired power of.95. The analysis indicated that a total sample size of 56 participants (approximately 19 per condition) would be sufficient to detect the expected effects (Pillai *V* = 0.61, actual power = 0.95). The final sample of 70 participants (attachment-insecurity priming *n* = 23, attachment-security priming *n* = 25, control condition *n* = 22) exceeded this requirement, ensuring adequate statistical power for detecting the hypothesized effects.

The initial sample included 75 participants, however, three of them were excluded from analyses due to computer failure and two more participants—due to self-reported difficulties in the priming task. In the sample of remaining 72 participants, two of them (3%) declared a problem in imaging and describing the targeted situation, both in the insecurity priming condition. Therefore, they were subsequently excluded from the sample. The remaining participants (final *N* = 70) declared that it was *definitely easy* (57%) or *just easy* (43%) to complete the priming procedure. These responses were evenly distributed across the priming conditions, χ^2^ [2, N = 70] = 3.46, *p* = 0.180, and *V* = 0.22. Furthermore, the biserial point correlation showed no significant links between these two categories of answers and attachment anxiety, *r* (68) = −0.07, *p* = 0.550, or attachment avoidance, *r* (68) = 10, *p* = 0.090. Hence, the final sample included 70 beginning undergraduate social sciences students from local university.

To minimize possible confounds, the participants were native Polish speakers; they had no vision deficits, no specific learning difficulties such as dyslexia, no motor control impairments, and all were right-handed. Furthermore, the subjects were not familiarized with the tasks used in the study. All were experienced computer and mouse users. Participation in this research was voluntary, and all participants provided informed consent. Participants were allowed to choose between a shopping voucher of the value of 30 PLN or credit points in their academic courses for completing the study.

### 3.2 Procedures and measures

#### 3.2.1 Experimental manipulation

Participants’ cognitive accessibility of attachment-related and non-attachment-related representations was manipulated using three different instructions. The primes were modeled on similar procedures used, among others, by Bartz and Lydon (2004 p. 1393), Gillath et al. (2009), and Troyer and Greitemeyer (2018).

In the three priming conditions, participants received written instructions. The specific instructions for the supraliminal attachment-security priming condition were as follows:

I would like to know about specific events and your thoughts and feelings about experiencing a good relationship with someone close to you (e.g., your mother, father, best friend, or romantic partner). Imagine that you ays rely on this person; she or he is close to you, accepts you, loves you, and helps you in times of need. You feel that this person cares for your well-being and supports you as best she or he can. In other words, she (he) is as reliable as possible.

In the supraliminal attachment-insecurity priming condition, participants received the following description:

We would like to know about specific events and your thoughts and feelings about experiencing an unsatisfactory relationship with someone close to you (e.g., your mother, father, best friend, or romantic partner). Imagine that you have not been able to count on this person for a long time. She or he is not always sensitive to your needs and does not support you as you would expect from someone close.

After reading the relationship description, the participants from the two attachment priming conditions were given the following instructions:

Now, take a moment and try to get a visual image of this person in your mind. Try writing down the feelings and thoughts that arise when you think of her or him in this way. You can describe specific, real, or imagined situations and events, your own and that person’s behavior, thoughts, feelings, and desires. Here are some helpful questions: What does this person look like? What is it like to be with this person? You may want to remember the time you were actually with this person. What would she or he say to you? What would you say in return? How do you feel when you are with this person? How would you feel if they were here with you now?

I also included the supraliminal control condition (no attachment priming) to provide a critical baseline against which both types of attachment priming can be compared. Furthermore, the control condition allows us to test whether any effects on non-attachment words are attributable to the experimental manipulations rather than unrelated factors, thereby strengthening causal inferences about the domain-specificity of attachment processes (Carnelley and Rowe, 2010; Jones et al., 2022). In this supraliminal control priming condition, participants received the following instruction:

We would like to know about specific events and your thoughts and feelings regarding your daily activities. Imagine you go to the store to buy things for your house. Think about that store’s location, then go through the shelves and the paths you have to go through, choosing the items you need. Imagine a typical shopping trip where everything is perfectly normal. Try writing down the feelings and thoughts that arise as you engage in this routine activity. You can describe specific, real, or imagined situations and events, as well as your behavior, thoughts, feelings, and desires.

During the priming task, the subjects wrote down their ideas on the blank sheet of A4, and the experimenter controlled the time. He stopped the task after 5 min, signaling it with a short, soft, and mellow stopwatch sound. At the end of the session, as a manipulation check, the participants were asked the following question: *“How easy it was to imagine (or recall) and describe the targeted situation?”* (see Procedure). The answers were assessed on a scale ranging from 1 (not at all) to 4 (definitely easy).

#### 3.2.2 The lexical decision task

Following the methodology of Mikulincer et al. (2000), a lexical decision task (LDT) was used to assess cognitive accessibility as a measure of the effects of the supraliminal attachment priming conditions on the accessibility of attachment-related representations (word categories). It was programed in the Psychology Experiment Building Language Version 2.1 (PEBL; Mueller and Piper, 2014) was used to program it and run it on a Dell PC with a 24-inch color monitor. The brightness and contrast were set to be low, and the letter strings were displayed in white on a black background in the middle of the screen. Participants were given 50 experimental trials and worked at their own pace. They were instructed to judge, as quickly and accurately as possible, whether each letter string was a valid Polish word by pressing the left (for word) or right (for nonword) *shift* keys on the keyboard. Each trial began with an “x” in the middle of the monitor, followed by a string of letters. After the participant responded, the letter string disappeared from the screen, followed by a 1,000 ms pause before the subsequent trial began. The primary dependent variables in this task were measured in milliseconds and computed as the average RTs for proximity and distance words. The lower the mean RT for a given word category, the higher the accessibility of the attachment-related representations (word categories).

All target stimuli were nouns, a decision consistent with prior attachment-priming research using lexical decision tasks (e.g., Mikulincer et al., 2000). Restricting the stimulus set to nouns reduced variability in morphological complexity and ensured greater control over psycholinguistic properties such as word length, frequency, and concreteness across categories.

The 50 letter strings were constructed in the same way as in the study of Mikulincer et al. (2000; Study 1 and Study 2), according to 6 categories. *Proximity words* were 5 Polish nouns that connote the attainment of proximity to others (i.e., “care,” “kisses,” “love,” “proximity,” “trust”). *Distance words* were 5 Polish nouns connoting interpersonal distance or the failure to attain proximity (i.e., “betrayal,” “contempt,” “distance,” “divorce,” and “separation*”*). *Positive words* were 5 Polish nouns with a positive connotation but no link to proximity themes (i.e., “award,” “beauty,” “joy,” “optimism,” “wisdom”). *Negative words* were 5 Polish nouns with a negative connotation but no link to proximity themes (i.e., “defeat,” “darkness,” “nightmare,” “troubles,” and “sadness*”*). Following Mikulincer et al. (2000), both positive and negative word categories were included to control for the possibility that the effects of attachment priming on the lexical decision task are due to the affective value of these words rather than to their attachment-related meaning. *Neutral words* were 5 Polish nouns with no salient positive or negative connotations and no link to proximity themes (i.e., “boat,” “box,” “desk,” “dishes,” “story”). This category was introduced to control for possible non-specific attachment priming effects on lexical decisions. *Nonwords* were 25-letter strings created from common Polish nouns (e.g., “oar” [pl., “wiosło”]) by changing 1–2 letters in random positions while retaining orthographic legality in the Polish language (e.g., “woisło*”*). Each word and nonword was presented once for a total of 50 trials. Trials were randomly ordered across participants. To disentangle the effects of word length and frequency on response latencies, I matched the stimuli for word length (i.e., 6 to 8 letters) and the Zipf word frequency indices^1^ (between 3.10 and 5.05 in each word category) derived from the SUBTLEX-PL database (Mandera et al., 2015). Hence, the stimuli categories did not differ significantly in terms of letter length (*F* (4, 20) = 0.00, *p* > 0.990, ηp^2^ = 0.00) or Zipf frequency indices, *F* (4, 20) = 0.42, *p* = 0.790, ηp^2^ = 0.08.

Following Mikulincer et al. (2000), in order to validate the categorization of the words, a sample of 10 psychologists rated the extent to which each of the above-described 25 words (a) reflected themes related to interpersonal relationships and (b) had a positive affective meaning. These ratings were performed on a 6-point scale ranging from *not at all* (1) to *very much* (6). Each participant’s ratings were averaged across the word categories, and analyses of variance (ANOVAs) for repeated measures were performed to examine differences between the word categories. These analyses revealed that proximity (*M* = 5.78, *SD* = 0.22) and distance (*M* = 5.32, *SD* = 0.80) words were rated as reflecting more interpersonal relationship themes than positive (*M* = 2.20, *SD* = 0.76), negative (*M* = 2.02, *SD* = 0.77), and neutral (*M* = 1.18, *SD* = 0.24) words, *F* (4, 36) = 140.31, *p* < 0.001, ηp^2^ = 0.94. Moreover, proximity (*M* = 5.81, *SD* = 0.21) and positive (*M* = 5.50, *SD* = 0.33) words were rated as having more positive meaning than neutral words (*M* = 2.72, *SD* = 0.52), which, in turn, were rated as having more positive meaning than distance (*M* = 1.64, *SD* = 0.47) and negative (*M* = 1.30, *SD* = 0.48) words, *F* (2.47, 22.23) = 317.74, *p* < 0.001, ηp^2^ = 0.97 (a Greenhouse–Geisser adjustment of the degrees of freedom was performed in anticipation of a sphericity assumption violation). These findings validated the categorization of the words used in this study.

#### 3.2.3 Attachment dimensions

Attachment dimensions were assessed using a Polish short version of Experiences in Close Relationships-Revised (ECR-RS; Lubiewska et al., 2016). This version contains eight items assessing attachment anxiety (e.g., *I worry about being abandoned*) and eight items assessing attachment avoidance (e.g., *I do not feel comfortable opening up to my loved ones*). Item answers form a 7-point Likert-type rating scale, ranging from 1 (*strongly disagree*) to 7 (*strongly agree*). In this measure, participants were asked to think about their experiences and feelings in close relationships without focusing on a specific one. The mean scores for each subscale were calculated following reverse scoring of negatively worded items, resulting in two continuous attachment anxiety and attachment avoidance indicators. The internal consistency of the Polish shortened ECR-R was *ω* = 0.77, *α* = 0.89 for anxiety, and ω = 0.80, α = 0.81 for avoidance (Lubiewska et al., 2016). In this study, those coefficients were: ω = 0.90, α = 0.90 for anxiety and ω = 0.84, α = 0.85 for avoidance.

#### 3.2.4 Felt-security state

Brief Felt-Security Scale (FSS), based on the work of Luke et al. (2012), was included as a phenomenological manipulation check, assessing the felt-security state as comprising the feelings of safety, love, care, and esteem. An 8-item version was constructed, and the participants indicated how they were currently feeling *cared for, comforted, loved, protected, safe, secure, supported,* and *valued*. Item answers are rated using a 6-point Likert-type rating scale, ranging from 1 (*strongly disagree*) to 6 (*strongly agree*). The total score was calculated by averaging the items. This study used McDonald’s omega and Cronbach’s alpha coefficients. They were: ω = 0.95, α = 0.95, respectively, indicating that the items formed a reliable scale.

#### 3.2.5 General trait anxiety

Since attachment anxiety and general trait anxiety share some variance but represent distinct constructs (e.g., Fraley et al., 2000), statistically controlling for trait anxiety ensures that any observed effects are attributable to attachment-specific mechanisms rather than general dispositions tendencies, the subtest measuring trait anxiety of the State–Trait Anxiety Inventory (STAI; Spielberger, 1983) in Polish adaptation of Wrześniewski et al. (2011) was used in the study. It is a 20-item scale that asks respondents to rate each item on a 1 (*not at all*) to 4 (*very much*) scale. The Trait Anxiety Subscale has adequate internal consistency reliability (Wrześniewski et al., 2011). In this study, the reliability coefficients were: ω = 0.95 and α = 0.89. Averaging items computed the score after appropriate reverse-scoring of designated items.

### 3.3 Procedure

The procedure was approved by the Ethics Committee of the SWPS University in Poznań (Poland) (decision no. 2022–127). Data collection took place between April 1, 2022, and June 1, 2022, with each participant completing the study procedures only once during this period. Invitation letters were sent to the students recruited on a volunteer basis. All participants provided written informed consent for their participation, and they could withdraw from the study at any time. The recorded data were analyzed anonymously.

Participants were individually tested in a 15-min experiment presented as a study of personality and word recognition. They were assured that their answers would remain anonymous and confidential. The experimenter was blind to the study hypotheses. Each session was started by presenting an outline of the experiment to familiarize participants with the procedure and signing an informed consent agreement.

Then, the participants were instructed on how to perform the lexical decision task and completed 10 training trials in which the words and non-words were non-attachment specific and differed from those in the experimental trials. Participants were informed that a similar task would appear later as one of the further procedures in this study. The practice trials were separated from the experimental trials in order to prevent erasing the effects of the priming conditions by the practice trials.

Following this introduction, the participants were randomly assigned to one of three priming conditions, using a computer-generated allocation sequence prepared prior to data collection. Assignment was implemented sequentially at the beginning of each experimental session, and the mental imagery priming task began. The supraliminal priming task was administered using fixed written instructions. The experimenter did not provide additional explanations or feedback during priming and was limited to time control.

Subjects completed the lexical decision task immediately after the priming task. They were instructed not to talk to the experimenter between the two tasks to avoid disturbing the experimental manipulation’s influence on the participants’ LDT performance. After completion of the LDT, they filled out the set of questionnaires, which were administered in a fixed order as follows: (1) Felt-Security Scale, (2) STAI-trait, (3) ECR-RS, and (4) a manipulation check asking participants a question: *How easy it was to imagine (or recall) and describe the targeted situation?*. The answers were assessed on a scale ranging from 1 (not at all) to 4 (definitely easy).

The fixed order of the questionnaires was used because the Felt-Security Scale served as another experimental manipulation check. Therefore, the shortest possible time distance between the priming task and the Felt-Security Scale was considered necessary to maintain the validity and reliability of the scale’s results. Furthermore, the ECR-RS was administered as the last measure as this assessment may activate chronic attachment-related representations during the priming task and/or during the Felt-Security Scale completion. Although all measures were completed within a single session, questionnaire responses were not inspected or scored until after data collection had been completed.

At the end of the study, participants underwent a standardized debriefing in which the experimenter asked whether they perceived any connection between the priming task and the lexical decision task or had formed any hypotheses about the study’s purpose. No participant indicated suspicion about the aims of the study or awareness of the link between the tasks.

### 3.4 Data preparation and statistical analyses

As part of data screening and cleaning, I focused on reaction times (RTs, in milliseconds) for *correct* lexical decisions. Trials with RTs below 250 ms—suggesting random responding—and above 2000 ms were excluded (Mikulincer et al., 2000). Fewer than 2% of all trials exceeded 1,500 ms, and no participant had more than 5% of trials above 2000 ms. Extreme RTs were additionally flagged using a standardized cut-off of *z* > 3.29 (Field, 2024) and accounted for < 5% of responses per participant, with a random distribution across word categories. Because these outliers were few, evenly distributed across conditions, and fell within plausible cognitive latencies, they were retained for analysis. Overall, the accuracy was high: the mean error rate was 3.7%, and every participant scored above 90%. Finally, one participant had missing data on the trait-anxiety subscale of the STAI-T; the missing values were replaced using person-mean imputation, which was considered adequate given the isolated nature of the missingness. The analyses were performed in IBM SPSS Statistics (version 29.0.2.0).

Descriptive statistics were calculated to summarize the data, and Pearson’s zero-order correlations were computed to examine the relationships between the dependent variables and the remaining variables and to determine their role as covariates. At this preliminary screening stage, assumptions of normality were acceptable at the univariate level for most variables; as expected for reaction time data, departures emerged at the multivariate level (George and Mallery, 2010; Tabachnick and Fidell, 2018).

The manipulation check was then conducted using a one-way analysis of variance (ANOVA) to examine the effect of the priming condition (security, insecurity, control) on state of felt-security. Partial eta-squared (η_p_^2^) values were calculated as measures of effect size, with values of 0.01, 0.06, and >0.14 representing small, medium, and large effects, respectively (Cohen, 1988). Bonferroni-adjusted *post hoc* tests were used to assess pairwise group differences. To quantify the magnitude of these differences, Hedges’ *g* was computed as the bias-corrected standardized mean difference. Consistent with Cohen’s (1988) guidelines, values of approximately 0.20, 0.50, and 0.80 were interpreted as small, medium, and large effects, respectively. 95% CI for Hedges’ *g* were derived using the standard error of the standardized mean difference.

A further set of ANOVA analyses was conducted to check whether attachment anxiety and avoidance scores were affected by priming conditions and whether general trait anxiety differed prior to experimental manipulation.

To evaluate the functioning of the control condition, raw reaction times to proximity- and distance-related words were compared within the control group using a conventional paired-samples *t*-test, complemented by a Two One-Sided Tests (TOST) equivalence procedure as a methodological robustness check. The equivalence bounds were set to *Δ* = ±40 ms, reflecting a conservative smallest effect size of interest (SESOI) defined in milliseconds at the level of the lexical decision task (Hutchison et al., 2013). The TOST equivalence analysis was conducted in jamovi (version 2.5.2). These preliminary checks were essential to confirm successful randomization, verify effective manipulation of attachment states, and ensure that observed priming effects were specific to attachment-related cognitive processes rather than influenced by pre-existing trait differences or general performance differences.

As each semantic category in the lexical decision task contained only five items presented once, the design may increase item-specific idiosyncrasies and reduce power to detect Condition × Category effects in aggregated RTs. To address this hierarchical structure and evaluate the robustness of subsequent aggregated analyses, I fitted a supplementary linear mixed-effects model (LMM) treating participants and items as crossed random effects, with random slopes for Category where identifiable. This approach accounts for trial-level variability and evaluates the robustness of aggregated findings to explicit modeling of item-level variance. Reaction times were log-transformed prior to modeling to reduce skewness and stabilize variance; reported estimates therefore reflect effects on log-transformed RTs and are not directly comparable in magnitude to effects expressed in milliseconds. All LMM analyses were conducted in R (version 2024.09.1 R Core Team, 2024) using the lme4, lmerTest, and emmeans packages.

Several assumptions of MANOVA were evaluated before proceeding with the main analysis. Univariate skewness and kurtosis were within ±2 for all dependent variables, indicating acceptable normality (George and Mallery, 2010), however, Mardia’s multivariate coefficients indicated some departure from multivariate normality, χ^2^ (165) = 265.42, *p*s < 0.05. Box’s M test revealed heterogeneity of covariance matrices, χ^2^ (30) = 60.88, *p* < 0.001, a pattern frequently observed in reaction time data. Given the balanced group sizes (22–25 per condition) and the superior robustness of Pillai’s Trace to violations of normality and homogeneity (Olson, 1976; Hair et al., 2022), Pillai’s *V* was selected as the omnibus MANOVA statistic. Pairwise Pearson correlations among the five reaction-time variables were below the 0.80 multicollinearity threshold, with largest *r* = 0.74 (Tabachnick and Fidell, 2018), and scatterplots confirmed linear relationships among dependent variables. Those primary inferential analyses reported in the main text were conducted on raw reaction times to preserve interpretability of latency differences in milliseconds, which remain central to the interpretation of processing efficiency in lexical decision paradigms (Fischler and Bloom, 1979).

To test the hypotheses, I used a two-step multivariate analysis plan that mirrors the order of my hypotheses. In the first step (focused model), MANOVA was used to test the main effect of the experimental condition (three levels) on reaction times for five word categories as dependent variables while controlling only for trait anxiety, thus addressing H1a–H1b. Significant multivariate effects were followed by univariate ANCOVAs and pairwise Bonferroni-adjusted comparisons. The partial eta-squared (ηp^2^) was interpreted as 0.01 = small, 0.06 = medium, and >0.14 = large effect size (Cohen, 1988). In the next step (comprehensive model), attachment anxiety and attachment avoidance were then added as covariates together with the interaction terms *Condition × Attachment Anxiety* and *Condition × Attachment Avoidance*, allowing me to examine moderation (H2a–H2b). This approach aligns with best practices in attachment research, where both direct experimental effects and individual differences contribute to understanding attachment system dynamics (e.g., Mikulincer et al., 2000).

Significant interaction effects involving attachment dimensions were further probed using regression-based moderation analyses. To analyze the significant interaction between the experimental condition and attachment dimensions, I employed the Hayes’ PROCESS macro for SPSS v. 4.2 (Model 1; Hayes, 2013, 2023). I used dummy-coded experimental conditions (with the control condition as reference). The moderator variable (attachment dimension) was mean-centered. The PROCESS analysis provided tests of conditional effects at relatively low and high levels of attachment dimensions (± 1SD). I reported unstandardized regression coefficients (b), standard errors, t-values, *p*-values, and 95% confidence intervals for these conditional effects. The increase in R^2^ due to the interaction (ΔR^2^) served as effect size measure, with values of 0.02, 0.13, and 0.26 representing small, medium, and large effects, respectively (Cohen, 1988).

Furthermore, to complement frequentist analyses, I conducted Bayesian ANCOVAs for each word category (proximity, distance, positive, negative, neutral), with Condition as a fixed factor and trait anxiety as a covariate. Those analyses, conducted on the same scale as the corresponding primary models (i.e., raw RTs), were implemented in jamovi (version 2.5.2) using model comparison against the null model (BF₁₀) and the *across matched models* option, which also provides inclusion Bayes factors (BF Inclusion) for each effect. Bayes factors were interpreted following Wagenmakers et al. (2018), where BF₁₀ > 3 indicates evidence for the presence of an effect and BF₀₁ > 3 indicates evidence for the absence of an effect. This approach allowed me to quantify the relative strength of evidence for Condition across semantic domains and to evaluate whether priming effects were selectively present for attachment-related categories.

Finally, to evaluate the robustness of the primary findings across different analytic assumptions, three sets of supplementary analyses were conducted. First, all primary MANOVA and ANCOVA models were re-estimated using log-transformed RTs to reduce skewness and assess whether the observed effects depended on the raw RT scale. These analyses indicated that the substantive pattern of significant and non-significant effects remained unchanged (see Supplementary materials S7.1). Second, trial-level accuracy data were analysed using a mixed-effects logistic regression with Condition and Word Category as fixed effects and crossed random intercepts for participants and items (Supplementary materials S7.2), allowing us to evaluate whether the RT effects could be attributed to speed–accuracy trade-offs rather than differences in lexical accessibility. Third, to address concerns about multiple testing across the five RT outcomes and to provide familywise control of false positives at the omnibus level, a permutation-based MANOVA (10,000 label shuffles) was conducted as a conservative sensitivity check of the multivariate Condition effect. This procedure yields a distribution-free p-value for the multivariate test by comparing the observed Pillai’s Trace to its permutation distribution. The permutation MANOVA was performed on log-transformed RTs with trait anxiety included as a covariate (Supplementary materials S7.3). Together, these supplementary analyses support the robustness of the category-specific findings to scale transformations and speed–accuracy trade-offs, while addressing familywise error control at the omnibus level. Full methodological details and statistical outputs are provided in Supplementary materials S7.1–S7.3 and in the OSF repository.

## 4 Results

### 4.1 Demographics

The final sample included 70 beginning undergraduate social sciences students from SWPS University in Poznan (73% female and 27% male) with an age range of 19–45 years (*Me* = 23.50; *M* = 25.03; *SD* = 5.43). Participants were randomly assigned to one of the three conditions. In the security priming condition, there were 25 participants (64% female; *M*_age_ = 24.68 years, *SD* = 4.42); in the activation of attachment system condition, there were 23 participants (70% women, *M*_age_ = 24.74 years, *SD* = 5.75); and in the control condition, there were 22 participants (64% women, *M*_age_ = 25.73 years, *SD* = 6.25). The three conditions did not differ significantly in sample sizes, χ^2^ (2) = 0.20, *p* = 0.91. There were no significant differences in participants’ age between experimental conditions, *F* (2, 67) = 0.261, *p* = 0.771, ηp^2^ = 0.01, and in gender distribution, χ^2^ (2) = 2.64, *p* = 0.273.

### 4.2 Descriptive statistics and correlations

First, descriptive statistics and Pearson zero-order correlations, both on raw RTs, were calculated for all study variables under the experimental conditions (Table 1). Absolute values of skewness (0.00–1.04) and kurtosis (−1.20–0.90) were within acceptable limits, suggesting that none of the variables markedly deviated from normality (George and Mallery, 2010).

**Table 1:** Descriptive statistics for main study variables across experimental conditions (*N* = 70).

Variable	Priming condition	*M*	*Me*	*SD*	Minimum	Maximum	Skewness	*SE*	Kurtosis	*SE*
Attachment anxiety	Insecurity^a^	3.64	3.63	1.45	1.00	7.00	0.39	0.48	−0.06	0.93
	Security^b^	3.48	3.50	1.60	1.00	6.25	0.09	0.46	−1.00	0.90
	Control^c^	3.36	3.25	1.34	1.00	5.25	−0.17	0.49	−0.86	0.95
Attachment avoidance	Insecurity	2.59	2.50	1.03	1.25	4.88	0.84	0.48	0.21	0.93
	Security	2.37	2.25	1.03	1.00	4.63	0.84	0.46	−0.12	0.90
	Control	2.42	2.13	1.10	1.00	5.00	0.86	0.49	−0.04	0.95
Felt security	Insecurity	2.47	2.38	0.74	1.00	4.00	0.23	0.48	−0.18	0.93
	Security	4.86	5.00	0.81	2.50	6.00	−1.46	0.46	2.61	0.90
	Control	3.48	3.56	0.94	2.00	5.38	0.05	0.49	−0.95	0.95
Trait anxiety	Insecurity	2.28	2.29	0.56	1.41	3.71	0.57	0.48	0.63	0.93
	Security	2.23	2.29	0.49	1.06	3.00	−0.57	0.46	−0.13	0.90
	Control	2.14	2.18	0.43	1.18	3.00	−0.05	0.49	0.14	0.95
Proximity words (ms)	Insecurity	729.95	723.25	107.36	546.00	931.20	0.10	0.48	−0.92	0.93
	Security	832.17	835.20	146.17	522.00	1194.20	0.32	0.46	0.57	0.90
	Control	887.52	879.00	199.76	579.40	1277.40	0.37	0.49	−0.61	0.95
Distance words (ms)	Insecurity	777.79	778.80	106.41	561.60	938.80	−0.25	0.48	−0.62	0.93
	Security	1025.00	998.60	287.81	562.80	1693.00	0.62	0.46	−0.11	0.90
	Control	961.27	935.80	249.12	568.40	1372.00	0.04	0.49	−1.15	0.95
Positive words (ms)	Insecurity	834.35	803.25	242.22	516.80	1579.80	1.84	0.48	3.83	0.93
	Security	958.09	893.00	246.07	575.40	1560.00	0.78	0.46	0.01	0.90
	Control	908.38	808.80	270.03	513.80	1558.80	0.88	0.49	0.22	0.95
Negative words (ms)	Insecurity	813.73	790.00	133.68	609.40	1218.00	1.20	0.48	2.71	0.93
	Security	914.90	875.75	183.73	579.80	1211.00	0.12	0.46	−1.16	0.90
	Control	912.02	859.00	262.04	564.25	1496.00	0.82	0.49	−0.04	0.95
Neutral words (ms)	Insecurity	869.37	858.25	181.30	585.80	1262.80	0.51	0.48	−0.10	0.93
	Security	950.87	875.80	239.66	609.00	1502.00	0.68	0.46	−0.51	0.90
	Control	1009.20	1000.10	273.44	578.60	1548.80	0.44	0.49	−0.47	0.95

^a^Attachment-insecurity priming (*n* = 23).

^b^Attachment-security priming (*n* = 25).

^c^Control condition (*n* = 22).

Biserial correlation analysis in the general sample are shown in Supplementary Table S1 revealed that age and sex were not linked to any of the remaining study variables; therefore, they were not included in the main analyses. The analysis also revealed that attachment avoidance and anxiety were moderately and positively correlated (*p* = 0.002). Attachment anxiety was strongly and positively related to general trait anxiety (*p* < 0.001) and weakly and negatively related to the mean RT for proximity-related words (*p* = 0.020). On the other hand, attachment avoidance was not linked to the general stat anxiety or the mean RTs for any of the five-word categories. Moreover, no significant links were found between the attachment dimensions and the felt-security state. However, the latter one was related weakly and positively to the mean RT for distance words (*p* = 0.040). Intercorrelations between mean RTs for words from all five semantic categories were moderate to strong and positive (all *p*s < 0.01) (for correlations across the three condition, see in the Supplementary Table S2).

### 4.3 Methodological controls and manipulation check

Before conducting the main analyses, I conducted some preliminary analyses to ensure the validity of my experimental paradigm.

#### 4.3.1 Manipulation check: felt-security

As a fundamental manipulation check, I expected that the supraliminal priming would modulate the participants’ state of felt-security, with security primes producing the highest levels, followed by control and then insecurity primes. To assess the effectiveness of my experimental manipulation, a one-way ANOVA was conducted with priming conditions (security, insecurity, control) as the between-subjects factor and state of felt-security as the dependent variable. As predicted, the priming condition had a significant effect on felt-security, *F* (2, 67) = 49.73, *p* < 0.001, ηp^2^ = 0.60. Bonferroni-adjusted *post hoc* comparisons revealed that participants in the attachment-security priming condition reported significantly higher felt-security (*for M*s and *SD*s within conditions, see Table 1) than those in the control (*p* < 0.001, Hedges *g* = 1.62, 95% CI [0.96, 2.29]) and insecurity priming (*p* < 0.001, *g* = −2.81, 95% CI [−3.62, −2.01]) conditions. The control group also reported a significantly higher sense of felt-security than the insecurity group (*p* < 0.001, *g* = −1.20, 95% CI [−1.84, −0.56]). All pairwise effects were large in magnitude, consistent with the substantial omnibus effect size. Full computational details and the R script used to derive these estimates are provided on the Open Science Framework: https://osf.io/y3j28/overview?view_only=24d5f4eabc9e49d4986318392d8c2771. Also, see Supplementary Table S5 for full results. The results confirm that attachment priming manipulation successfully altered participants’ subjective sense of relational security, with increased security priming increasing and decreased insecurity priming decreasing feelings of felt-security. This finding validates the effectiveness of the experimental manipulation.

#### 4.3.2 Priming conditions, attachment dimensions, and general trait anxiety

A one-way ANOVA comparing the priming conditions showed no significant differences in attachment anxiety, *F* (2, 67) = 0.211, *p* = 0.810, ηp^2^ = 0.01, attachment avoidance, *F* (2, 67) = 0.28, *p* = 0.759, ηp^2^ = 0.01 and general trait anxiety, *F* (2, 67) = 0.43, *p* = 0.652, ηp^2^ = 0.01. Thus, participants did not differ across the priming conditions in terms of attachment dimensions and general trait anxiety.

#### 4.3.3 Control condition—reference status assessment

Within the control condition, raw RTs to proximity- and distance-related words were compared using a conventional paired-samples *t*-test. No statistically significant difference was observed (for condition-specific means and standard deviations, see Table 1), *t* (21) = −1.79, *p* = 0.087, *d* = −0.04, 95% CI [−0.81, 0.05]. RTs to proximity and distance words were strongly correlated, *r* (21) = 0.65, *p* = 0.001, indicating substantial within-person consistency in participants’ response patterns across attachment-related word categories.

However, because non-significant results do not constitute affirmative evidence of equivalence, a Two One-Sided Tests (TOST) equivalence procedure was conducted as a methodological robustness check, with equivalence bounds set to *Δ* = ±40 ms on the raw RTs. Equivalence testing did not support statistical equivalence between proximity- and distance-related reaction times in the control condition (lower test: *t* (21) = 2.77, *p* = 0.006; upper test: *t* (21) = 0.82, *p* = 0.79). The observed standardized mean difference was small (Hedges’ *g* = 0.20, 90% CI [−0.15, 0.57]). Accordingly, although the proximity–distance contrast was not statistically significant under conventional significance testing, the control condition is interpreted as a non-primed reference condition rather than as a formally validated neutral baseline.

#### 4.3.4 Trial-level robustness analysis (LMM)

Before reporting the primary hypothesis-driven analyses, a supplementary trial-level robustness check was conducted to assess the sensitivity of aggregated reaction-time results to item-level variability and distributional skewness. Specifically, a supplementary linear mixed-effects model (LMM) was fitted with participants and items treated as crossed random effects and with random slopes for Category where identifiable. Reaction times were log-transformed to reduce positive skewness—a variance-stabilizing transformation that improves model assumptions but can reduce sensitivity to additive effects expressed in milliseconds. This modeling approach accounts for item-specific variance that cannot be examined in ANOVA-based frameworks.

The model revealed no significant main effect of Condition, *F* (2, 62.8) = 1.47, *p* = 0.232, no main effect of Category, *F* (4, 24.2) = 2.03, *p* = 0.125, and no Condition × Category interaction, *F* (8, 1,396) = 0.14, *p* = 0.983. Bonferroni-adjusted pairwise contrasts were also nonsignificant. Taken together, these results indicate that explicit modeling of item-level variability did not yield effects in directions contradicting the aggregated pattern of findings. At the same time, power to detect Category effects at the trial level was limited by the small number of stimuli per semantic category.

Full fixed-effect estimates, variance components, and Bonferroni-adjusted pairwise contrasts are reported in Supplementary Table S6, and the complete R script is available in the OSF repository (see Data availability statement).

### 4.4 Main analyses

Unless otherwise stated, reported inferential statistics are based on analyses conducted on raw reaction times; results from log-transformed reaction time analyses are reported as complementary checks.

#### 4.4.1 Focused model: main effect of condition (H1a, H1b, and H3)

Controlling for trait anxiety, the multivariate test for condition was significant, Pillai’s V = 0.36, *F* (10, 114) = 2.49, *p* = 0.010, ηp^2^ = 0.18. Follow-up univariate ANCOVAs (Table 2) showed significant effects for proximity and distance words; other word categories were non-significant (*ps* ≥ 0.15). The estimated marginal means and standard errors are provided in the Supplementary Table S3. This pattern of results supports H3, which states that attachment priming specifically targets relational representations (the domain-specificity of attachment manipulations). The large effect sizes for attachment-related words (ηp^2^ > 0.14) compared with the small to medium effect sizes for non-attachment words (ηp^2^ between 0.05 and 0.06) further highlight the domain-specificity of attachment manipulations. Notably, the trial-level LMM did not reveal evidence suggesting that the aggregated effects were driven by idiosyncratic item variability, as no Condition effects emerged when modeling trial-level noise directly (see Supplementary Table 6).

**Table 2:** Univariate follow-up ANCOVAs of lexical decision times by priming condition controlling for trait-anxiety.

Dependent variable	*F*	*df1*	*df2*	*p*	ηp^2^
Proximity words	5.26	2	60	0.008	0.15
Distance words	7.07	2	60	0.002	0.19
Positive words	1.58	2	60	0.215	0.05
Negative words	1.94	2	60	0.152	0.06
Neutral words	1.78	2	60	0.177	0.06

To ensure that these findings did not depend on preprocessing decisions, all multivariate and univariate analyses were repeated using log-transformed RTs (see Supplementary materials S7.1). The transformation improved normality and yielded the same substantive conclusion that condition effects remained confined to proximity and distance categories.

In response to concerns about familywise Type I error arising from multiple dependent variables, a permutation-based MANOVA was conducted as a conservative sensitivity check of the omnibus multivariate Condition effect (10,000 label shuffles). This analysis did not yield a significant global multivariate effect (Pillai’s Trace = 0.10, *p*_perm = 0.82; see Supplementary materials S7.3). Given the conservative nature of permutation testing in small samples with multiple outcomes, this result is interpreted as reflecting limited power at the omnibus level rather than contradicting the category-specific effects observed in the primary and log-transformed analyses.

To further differentiate attachment-related from non-attachment-related processing, I run Bayesian ANCOVAs for each word category, with Condition as a factor and trait anxiety as a covariate. For attachment-related categories, the Bayes factors indicated strong evidence for a *Condition* effect (proximity: BF₁₀ = 10.38; distance: BF₁₀ = 27.19). In contrast, for non-attachment categories (positive, negative, neutral), the data consistently favored the null model (BF₀₁ = 1.82–2.78; BF₁₀ = 0.36–0.55), providing anecdotal evidence against a Condition effect. This marked asymmetry in Bayes factors constitutes affirmative evidence that priming effects on non-attachment categories were meaningfully smaller than those on attachment-related categories, consistent with domain-specific activation of attachment-related representations (see Supplementary Table S4 for full results).

Because RT differences could theoretically arise from differences in accuracy, trial-level accuracy data were analyzed using a mixed-effects logistic model (*n* = 63). Accuracy was uniformly high across cells (*M*s = 0.91–1.00), resulting in quasi-separation and no significant fixed effects (all *p*s > 0.22). Summary statistics are provided in Supplementary material S7.2. A speed–accuracy check showed no relationship between participants’ mean RTs (ms) and accuracy, *r* (61) = −0.02, 95% CI [−0.27, 0.23], *p* = 0.88, ruling out a speed–accuracy trade-off.

In pairwise comparisons, for proximity words (H1a), participants in the insecurity priming condition showed faster RTs than those in the non-primed reference condition (*Δ* = −143 ms, *p* = 0.007; 95% CI [−252.73; −32.49]). However, participants in the security priming condition (*M* = 827.31 ms, *SE* = 29.81) did not differ significantly from those in either the non-primed reference condition (*p* = 0.735, 95% CI [−158.90; 56.30]) or insecurity priming condition (*p* = 0.116, 95% CI [−14.98; 197.60]). These findings partially support H1a, as participants in the insecurity priming, but not in the security priming, showed faster RTs to proximity-related words compared to the non-primed reference condition.

For distance words (H1b), participants in the insecurity priming condition were faster than those in the security priming condition (Δ = −245 ms, *p* = 0.001, 95% CI [−408.38; −81, 68]) and those in the non-primed reference condition (Δ = −170 ms, *p* = 0.049, 95% CI [−339.28; −0.81]). On the other hand, no significant difference was found between the security priming and the non-primed reference conditions, *p* = 0.806, 95% CI [−90.38; 240.35]. These findings fully support H1b, participants in the insecurity priming condition responded significantly faster to distance-related words relative to both security priming and the non-primed reference condition.

#### 4.4.2 Comprehensive model: moderation by attachment dimensions (H2a and H2b)

Regarding hypotheses H2a and H2b, when examining the complete MANOVA model (on raw RTs) including individual differences in attachment style, the overall condition effect was no longer significant (Table 3). Similarly, no significant interaction emerged for *Condition x Attachment avoidance*. However, the Condition × Attachment anxiety interaction was observed at the trend-level (*p* = 0.058, ηp^2^ = 1.14). Althought it did not approach conventional significance, I conducted univariate ANCOVAs to identify the outcomes that contributed to this trend (Table 4). Indeed, a significant interaction emerged only for proximity-word latencies; all other word categories were non-significant (*p*s ≥ 0.17).

**Table 3:** Multivariate tests of condition with attachment orientations and their interactions, controlling for trait anxiety.

Effect	Pillai *V*	*F*	*df1*	*df2*	*p*	ηp^2^
Condition	0.19	1.21	10	114	0.294	0.10
Attachment Anxiety	0.06	0.77	5	56	0.578	0.06
Attachment Avoidance	0.14	1.75	5	56	0.138	0.14
Trait Anxiety	0.04	0.44	5	56	0.821	0.04
Condition × Attachment Anxiety	0.28	1.86	10	114	0.058	0.14
Condition × Attachment Avoidance	0.14	0.86	10	114	0.571	0.07

**Table 4:** Interaction effects of priming condition and attachment anxiety on lexical decision times across five word categories.

Dependent variable	*F*	*df1*	*df2*	*p*	ηp^2^
Proximity words	3.36	2	60	0.042	0.10
Distance words	1.29	2	60	0.283	0.04
Positive words	0.55	2	60	0.550	0.02
Negative words	1.90	2	60	0.159	0.06
Neutral words	2.12	2	60	0.129	0.07

Models control for trait anxiety.

Because attachment anxiety was strongly correlated with trait anxiety (*r* (68) = 0.66, *p* < 0.001), I examined the extent to which their overlap might account for the moderation findings before interpreting them. Attachment anxiety showed a moderate zero-order association with proximity-word RTs (*r* (68) = −0.28, *p* = 0.02). However, after controlling for trait anxiety, the association was attenuated and no longer statistically significant, although it remained in the same direction (partial *r* = −0.19, *p* = 0.13). Thus, the data provide limited evidence for a unique bivariate association between attachment anxiety and proximity-word RTs beyond general trait anxiety.

At the same time, the absence of a robust partial correlation does not imply redundancy between the two constructs at the model level. Collinearity diagnostics indicated acceptable separation between attachment anxiety and trait anxiety: variance inflation factors were low for both attachment anxiety (VIF = 1.79) and trait anxiety (VIF = 1.78), suggesting no problematic multicollinearity. Because trait anxiety was already controlled for in the primary models, these supplementary diagnostics provide additional reassurance that attachment anxiety captures variance that is not merely a statistical artifact of general anxiety.

**Figure 1:**
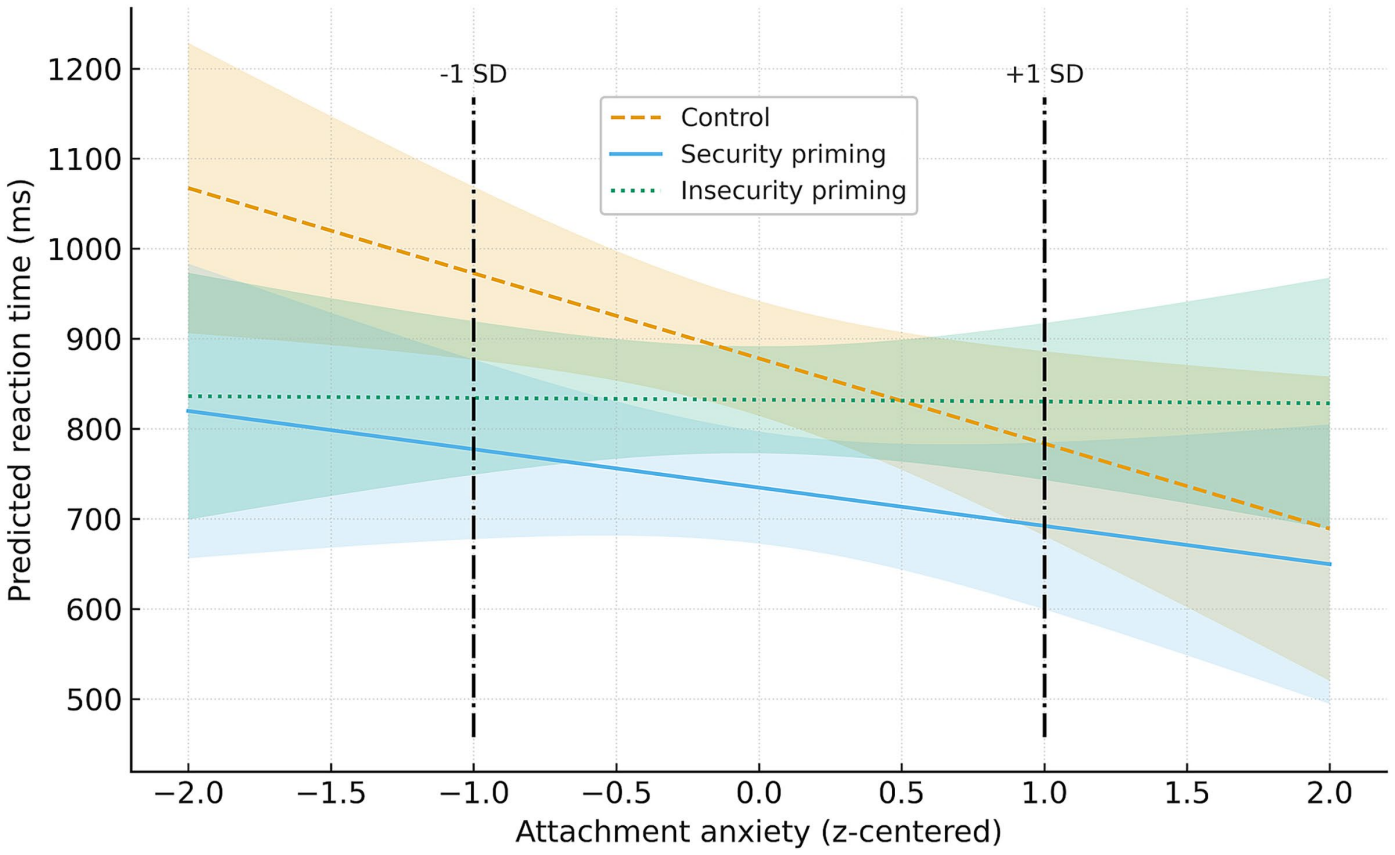
Interaction of priming condition and attachment anxiety in predicting proximity-word reaction times. The non-primed reference condition (referred to as *Control* in the figure) was used as the reference category in the moderation model (dummy coding). Plotted slopes therefore represent model-estimated conditional effects for each priming condition. Lines depict predicted reaction times with trait anxiety held at the sample mean, and shaded color bands represent 95% CIs. Line styles correspond to security (solid), reference (dashed), and insecurity (dotted). Vertical dash-dot lines indicate −1 SD and +1 SD on centered attachment anxiety, which were used to probe the Security × Anxiety interaction. As reported in the results section, the simple slope of security priming was significant at low attachment anxiety but not at high attachment anxiety.

To further examine aforementioned interaction effect, I conducted a moderation analysis using the PROCESS macro (Model 1; Hayes, 2013, 2023), with priming condition as a categorical predictor (dummy coded using the non-primed reference condition as the reference group), reaction time for proximity-related words (raw scores) as the outcome variable, attachment anxiety as the moderator, and trait anxiety as a covariate. The overall model was significant, *R^2^* = 0.27, *F* (6, 63) = 3.79, *p* = 0.003. A significant interaction was confirmed between the security vs. reference condition contrast and attachment anxiety (see Table 5, Model 1), with a small-to-medium effect size (∆*R*^2^ = 0.05). To interpret this interaction, I examined the conditional effects of security priming at low (−1 SD) and high (+1 SD) levels of attachment anxiety (Figure 1). At low attachment anxiety (−1.456), security priming condition had significantly faster reaction times to proximity-related words compared with the non-primed reference condition, *b* = −138.57, *SE* = 60.50, *t* = −2.29, *p* = 0.025, 95% CI [−259.46; −17.67]. At high attachment anxiety (+1.456), the effect of security priming was not significant, *b* = 46.63, *SE* = 64.49, *t* = 0.72, *p* = 0.472, 95% CI [−82.24; 175.50].

**Table 5:** Moderated regression predicting reaction times to proximity words across priming conditions (dummy-coded comparisons).

Contrast (Predictor)	*B*	*SE*	*t*	*p*	95% *CI*
*Model 1*					
Security vs Reference	−45.97	43.69	−1.05	0.297	[−133.27; 41.33]
Insecurity vs Reference	−143.47	44.70	−3.21	0.002	[−232.79; −54.15]
Attachment Anxiety	−64.97	26.18	−2.48	0.016	[−117.28; −12.66]
Security × Anxiety (vs Reference)	63.61	30.73	2.07*	0.043	[2.2; 125.02]
Insecurity × Anxiety (vs Reference)	35.75	32.74	1.09	0.279	[−29.6;, 101.17]
*Model 2*					
Security vs Insecurity	97.50	43.07	2.26	0.027	[11.43; 183.58]
Control vs Insecurity	143.47	44.70	3.21	0.002	[54.15; 232.79]
Attachment Anxiety	−29.22	25.23	−1.16	0.251	[−79.64; 21.20]
Security × Anxiety (vs Insecurity)	27.86	29.07	0.96	0.342	[−30.23; 85.96]
Reference × Anxiety (vs Insecurity)	−35.75	32.74	−1.09	0.279	[−101.17; 29.67]

Priming condition was dummy-coded in two models. In Model 1, the reference category was the non-primed reference condition; in Model 2, it was the insecurity priming condition. Contrast labels (e.g., “Security vs Reference”) indicate which group was coded 1 versus the reference group coded 0. Interaction terms reflect the moderation of priming effects by attachment anxiety. Model 1: R^2^ = 0.27, *F* (6, 63) = 3.79, *p* = 0.003. Model 2: *R*^2^ = 0.27, *F* (6, 63) = 3.79, *p* = 0.003.

Taken together, the Condition × Attachment Anxiety interaction followed the hypothesized direction but remained at the trend level when trait anxiety was included as a covariate. Accordingly, this effect did not reach conventional statistical significance in the more rigorous control model. Therefore, Hypothesis H2a was statistically supported at the trend-level in the model controlling for trait anxiety.

A complementary moderation analysis was conducted with the insecurity priming condition as the reference group, allowing for direct comparison between security and insecurity, as well as non-primed reference condition and insecurity (see Table 5, Model 2). However, the interactions between attachment anxiety and security vs. insecurity, and the contrasts between insecurity and the reference condition did not approach significance.

On the other hand, contrary to Hypothesis 2b, no significant interaction between attachment dimensions and condition on distance-related words was observed (Tables 3, 4). Likewise, no significant interactions emerged for any of the non-attachment word categories—positive, negative, or neutral words (all *Fs* ≤ 1.90, all *ps* ≥ 0.13).

## 5 Discussion

This study investigated the domain-specific effects of supraliminal attachment priming on the cognitive accessibility of attachment-related mental representations. The findings revealed a pattern in which experimental condition differentiated processing of attachment-related concepts, whereas no such differentiation was observed for non-attachment concepts, supporting the domain specificity of attachment processes. Below, I discuss the findings in relation to each hypothesis and their theoretical implications.

### 5.1 Domain-specific effects of attachment priming

Before evaluating the main hypotheses, I implemented several methodological controls to ensure the validity of my findings. First manipulation check confirmed that participants in the supraliminal security priming condition reported a significantly higher subjective sense of security than those in the non-primed reference condition, supporting the validity of the experimental manipulation. Results from the second methodological robustness check suggest that equivalence between proximity- and distance-related words was not demonstrated in the control condition. This finding implies that the control condition does not provide evidence of strict baseline neutrality but instead serves as a non-primed reference point against which attachment-related shifts in lexical accessibility can be evaluated.

Against the non-primed reference condition, the results nonetheless revealed a robust domain-specific organization, with experimental manipulations yielding reliable differences exclusively for attachment-related concepts (proximity and distance), while no such differences were observed for positive, negative, or neutral words. Importantly, all results were independent of general trait anxiety, highlighting the specificity of these effects to attachment-related processes rather than general emotionality. Moreover, the observed reaction time differences cannot be attributed to speed–accuracy trade-offs. Accuracy was uniformly high across conditions and unrelated to individual differences in response latency, supporting the interpretation of RT effects as reflecting genuine differences in the accessibility of attachment-related representations. Thus, guided-imagery supraliminal priming used in this study appears to selectively engage attachment-relevant cognitive networks, rather than producing a generalized affective facilitation. This pattern is consistent with attachment theory, which postulates that the attachment system operates as a distinct functional system with specialized cognitive processes (Bowlby, 1969; Mikulincer and Shaver, 2007a). The theoretical coherence of this domain-specific interpretation is particularly important given the supraliminal nature of the manipulation, as it supports the conclusion that the observed differences are attributable to attachment-specific processes rather than to generalized affective responses to consciously processed emotional stimuli. At the methodological level, supplementary trial-level analyses did not reveal evidence that the aggregated findings were driven by idiosyncratic item variability (see Supplementary Table 6).

### 5.2 Effects on proximity-related concepts (H1a and H2a)

Predictions concerning proximity-related accessibility were derived from the assumption that supraliminal attachment cues modulate the activation of secure-base representations. Accordingly, insecurity-related effects were expected to be more robust, whereas security-related effects were expected to be more conditional under supraliminal processing. I hypothesized that both supraliminal attachment insecurity and security priming would increase the cognitive accessibility of proximity-related words compared with the non-primed reference condition, as indicated by faster reaction times (RTs) in the LDT for proximity words (H1a). Consistent with part of this hypothesis, participants in the insecurity priming condition responded significantly faster to proximity-related words than those in the non-primed reference condition. This pattern is consistent with the interpretation of faster LDT responses as reflecting higher cognitive accessibility (Fischler and Bloom, 1979) and with attachment theory’s prediction that insecurity activation increases accessibility of proximity-related representations (Bowlby, 1969, 1982; Mikulincer and Shaver, 2003). Prior lexical decision studies using threat/stress primes similarly reported heightened access to proximity-related words (Mikulincer et al., 2000, 2002). This effect is considered a normative component of the stress-attachment link, reflecting activation of the attachment system under threat and increased accessibility of representations related to seeking proximity and support (Mikulincer et al., 2000). The large effect size observed for the difference between the insecurity priming condition and non-primed reference condition further underscores the robustness of this effect.

In contrast to this normative insecurity effect, participants in the supraliminal attachment security priming condition did not show uniformly faster reaction times to proximity-related words than those in the non-primed reference condition, as predicted in the second part of H1a. Instead, the pattern of differences between the security priming condition and the non-primed reference condition varied across levels of chronic attachment anxiety, with trait anxiety controlled in all analyses. Specifically, among individuals reporting low levels of attachment anxiety, reaction times to proximity-related words were significantly faster in the security priming condition than in the non-primed reference condition. This pattern is theoretically consistent with accounts proposing that security-related representations are associated with more efficient processing of safety-congruent information, support, and closeness when such representations align with individuals’ chronic attachment expectations (e.g., Hudson and Fraley, 2018; Mikulincer and Shaver, 2020). Although this pattern of differences was consistent with the hypothesized direction, the corresponding Condition × Attachment Anxiety interaction term reached only trend-level significance in the trait-anxiety–controlled model and did not reach conventional statistical significance. Accordingly, this result should be interpreted cautiously. Given the substantial overlap between attachment anxiety and general trait anxiety, statistically disentangling their unique contributions is challenging, particularly in samples of modest size; therefore, the present moderation findings should be considered suggestive rather than conclusive.

The findings further indicated that, at high levels of attachment anxiety, reaction times to proximity-related words did not differ significantly between the security priming condition and the non-primed reference condition. Thus, the relative advantage observed at low levels of attachment anxiety was absent among more anxiously attached individuals. This pattern aligns with prior work suggesting that the impact of security priming on anxiously attached individuals can be complex and not always straightforwardly positive (Mikulincer and Sheffi, 2000; Mallinckrodt et al., 2013; Long et al., 2020). Individuals high in attachment anxiety are characterized by hyperactivating strategies involving heightened monitoring of attachment-related cues and persistent concerns about abandonment and rejection (Mikulincer and Shaver, 2003). Although anxious individuals may generally show high accessibility of proximity-related thoughts regardless of context (e.g., Mikulincer et al., 2000), the present findings indicate that the relative advantage of the supraliminal security priming condition over the non-primed reference condition in proximity-word processing was not observed at higher levels of attachment anxiety.

This conditional pattern can be understood within established models of attachment-related regulation (Baldwin et al., 1996; Mikulincer and Shaver, 2003). Supraliminal security cues activate secure-base representations in a manner that allows conscious elaboration and regulatory engagement, thereby enabling individual differences in attachment-related strategies to shape subsequent cognitive processing (Mikulincer and Shaver, 2007a). For individuals low in attachment anxiety, security-related representations are likely to be schema-congruent, aligning with stable expectations of availability and responsiveness and allowing proximity-related concepts to be accessed with minimal regulatory effort (Zhang et al., 2025). In contrast, for individuals high in attachment anxiety, supraliminal security priming may introduce a mismatch between activated security representations and chronically accessible expectations of inconsistency, rejection, or abandonment (Mallinckrodt et al., 2013; Long et al., 2020). This mismatch can engage hyperactivating regulatory processes, resulting in ambivalence-related processing costs during lexical decision performance. Importantly, this interpretation of ambivalence-related processing costs should be understood as a theoretical inference derived from behavioral performance patterns rather than as a directly observed cognitive mechanism. Reaction times index the final output of processing efficiency rather than the moment-to-moment dynamics of cognitive conflict or regulatory engagement. Although no direct analyses of reaction time variability were conducted, supplementary trial-level mixed-effects models indicated that the observed effects were not driven by increased response noise or item-specific variability, supporting the interpretation of systematic performance modulation rather than response instability.

In contrast to the findings for attachment anxiety and the second part of Hypothesis 2a, analyses did not provided evidence for differences between the security priming condition and the non-primed reference condition in proximity-word reaction times as a function of chronic attachment avoidance. However, these null findings should be interpreted cautiously and considered exploratory. The absence of avoidance-related differences may reflect the deactivating regulatory strategies characteristic of avoidant attachment, which involve suppression or disengagement from attachment-relevant information (Andriopoulos and Kafetsios, 2015). Such strategies may be more likely to manifest under conditions of reduced conscious control, such as subliminal priming or increased cognitive load (Mikulincer et al., 2000; Han and Psouni, 2025; Zhang et al., 2025). The use of supraliminal priming without additional cognitive demands in the present design may therefore have limited sensitivity to avoidance-related modulation of proximity-word processing. Future research could examine whether avoidance-related differences emerge under alternative priming conditions or tasks involving higher regulatory demands.

Taken together, the obtained findings suggest that proximity-related representations are selectively modulated by security cues, whereas distance-related representations are more directly shaped by attachment insecurity.

### 5.3 Effects on distance-related concepts (H1b and H2b)

Predictions concerning distance-related accessibility were based on attachment theory’s assumption that insecurity activates withdrawal- and separation-related representations as part of a normative threat response. Consistent with this *a priori* calibration of the hypotheses, insecurity-related effects on distance representations were expected to emerge reliably, whereas security-related effects were not expected to produce systematic modulation of distance-word accessibility. Hypothesis H1b predicted that attachment insecurity priming would increase the cognitive accessibility of distance-related words compared to both the non-primed reference condition and the security priming condition, as indicated by faster reaction times. Consistent with this hypothesis, the results strongly supported H1b, demonstrating that participants in the insecurity priming condition responded significantly faster to distance-related words than those in both the non-primed reference and security priming conditions. In contrast, reaction times to distance-related words in the security priming condition did not differ significantly from those observed in the non-primed reference condition. The magnitude of this between-condition contrast was large, underscoring the robustness of the insecurity priming pattern for distance-related representations. Notably, this pattern contrasts with the proximity-related findings, which showed a more conditional response to security priming. When attachment dimensions were included in the comprehensive model, neither the overall condition effect nor interactions with attachment anxiety or avoidance reached conventional levels of statistical significance. Accordingly, these analyses should be interpreted as exploratory. Nonetheless, the large effect size for the insecurity priming effect on distance-word RTs justifies further consideration of these patterns, while clearly acknowledging their exploratory nature.

The lack of a significant difference between security priming and the non-primed reference condition for distance-related words is also noteworthy. Prior research suggests that security priming activates attachment-related representations associated with safety and support, which can shape subsequent cognitive processing without necessarily producing generalized affective facilitation (Carnelley and Rowe, 2010; Rowe et al., 2020; Gillath et al., 2025). However, in the present study, such activation did not translate into altered accessibility of distance-related concepts relative to the non-primed reference condition. This pattern may indicate that security primarily buffers against cognitive consequences of threat or insecurity rather than actively downregulating the accessibility of concepts of relational distance in a non-threatening context. Concerns about distance or separation may be therefore less chronically or contextually activated in a secure state than under insecurity, resulting in processing latencies comparable to those observed in the non-primed reference condition. In contrast, the substantial difference between insecurity and security priming conditions highlights the potential role of insecurity in selectively elevating the accessibility of separation-related concepts.

Furthermore, consistent with the exploratory analyses, attachment orientations did not modulate the effect of priming on distance-word accessibility. At first glance, this pattern may appear to contrast with some findings in the attachment literature. Mikulincer et al. (2000), using a subliminal priming approach, demonstrated that individuals with high attachment anxiety tend to show heightened accessibility to words related to proximity worries (such as “separation”), regardless of the priming context. On the other hand, individuals with high attachment avoidance are often described as employing deactivating strategies to manage attachment-related distress (Mikulincer et al., 2004, 2009). Various studies have found that avoidance moderates responses to attachment-related stimuli and priming. For instance, some studies found that highly avoidant individuals showed longer RTs to negative emotion words in an emotional Stroop task, particularly under supraliminal conditions (e.g., Andriopoulos and Kafetsios, 2015). However, these effects are highly task- and context-dependent. Thus, the effects of avoidance-related defenses appear to be task-dependent and are often more evident under subliminal priming or conditions involving cognitive load that limits suppressive control (Han and Psouni, 2025; Mikulincer et al., 2000). In the present lexical decision task, which involved supraliminal priming and did not impose additional cognitive load, attachment avoidance did not emerge as a significant moderator of distance-word accessibility. Although avoidance plays a well-established role in shaping responses to attachment-related cues, this null moderation finding should be interpreted cautiously, given the exploratory nature of the moderation analyses and the absence of a reliable main effect of avoidance on reaction times.

It is worth noting that this pattern contrasts with the proximity-related findings. For proximity-related words, a significant difference relative to the non-primed reference condition was observed for insecurity priming, whereas responses in the security priming condition varied as a function of attachment anxiety. For distance-related words, participants in the insecurity priming condition showed significantly faster reaction times compared with both the non-primed reference condition and the security priming condition. This pattern suggests that distance-related concepts are particularly responsive to the activation of attachment insecurity.

### 5.4 Differentiated model of attachment-related cognitive accessibility

Taken together, the present findings support a differentiated model of attachment-related cognitive accessibility under supraliminal processing. Insecurity priming produced robust and theoretically consistent facilitation of both proximity- and distance-related representations, whereas security priming effects were more conditional and dependent on individual differences in attachment-related regulation. Importantly, this asymmetry was anticipated based on the differential empirical grounding of the hypotheses and reflects the distinct functional roles of insecurity- versus security-related activation within the attachment system. By directly comparing supraliminal security, insecurity, and non-primed reference conditions within a single lexical decision paradigm, the present study clarifies how distinct modes of attachment system activation shape early accessibility of core relational representations.

### 5.5 Supraliminal vs. subliminal priming in attachment research

These differentiated patterns of security- and insecurity-related accessibility are particularly informative when considered in relation to the level of awareness at which attachment cues are processed. The use of a guided imagery task aligns with established supraliminal priming methods employed in attachment research, which often involve visualizing supportive figures or recalling secure experiences, as well as methods using pictorial representations or word sets (Rowe et al., 2020). The observed domain-specific influence of supraliminal attachment priming—which affects attachment-related mental representations without altering non-attachment concepts—is consistent with previous research demonstrating that attachment priming effects, whether subliminal or supraliminal, tend to be specific to the attachment domain (Mikulincer et al., 2000; Jones et al., 2022; Gillath et al., 2025; Lisá et al., 2025). Specifically, the present finding that supraliminal insecurity priming reliably increased the accessibility of proximity-related concepts aligns with findings from studies using both subliminal and supraliminal threat or insecurity priming. This convergence suggests that insecurity cues robustly activate attachment-relevant representations across levels of awareness. Additionally, in in an exploratory moderation analysis, individual differences in attachment avoidance and anxiety were found to influence responses to supraliminal security priming. Notably, this effect differed from the patterns previously observed with subliminal priming methods. Taken together, these findings contribute to a broader methodological perspective on attachment priming, highlighting how the level of awareness at which attachment cues are processed shapes the cognitive consequences that can be observed. Subliminal and supraliminal approaches appear to access distinct aspects of attachment system functioning: subliminal methods tap into automatic, less defensively-filtered processes (Mikulincer et al., 2002), whereas supraliminal techniques engage conscious, controlled, and arguably more ecologically valid processes (Carnelley and Rowe, 2010).

### 5.6 Limitations and future directions

Following recommendations for transparent reporting (Simons et al., 2017), I acknowledge several constraints that limit the generalizability of these findings. The results are most directly applicable to young adults (ages 19–45, *M* = 25.03) from university populations who are native Polish speakers, right-handed, and without vision deficits or learning difficulties. The sample was predominantly female (73%) and recruited from a single university in Poland, limiting generalizability across gender, cultural, and educational contexts. While the sample size was adequate for detecting main effects, it may have been insufficient to reliably detect subtle interaction effects, particularly those involving attachment avoidance. Some observed trends in moderation analyses, particularly involving attachment anxiety and avoidance, did not reach conventional levels of statistical significance. These findings should therefore be interpreted as exploratory, and future studies with larger samples are needed to confirm or clarify these potential effects. Future research should employ larger, more diverse samples to thoroughly explore these potential moderating effects and enhance generalizability across populations.

The methodological constraints further limit generalizability. The guided imagery priming methodology and lexical decision task were conducted in Polish, with attachment-related word stimuli specifically validated for Polish language and culture. The use of a single supraliminal priming method limits generalizability to other supraliminal techniques. Future studies should employ various supraliminal approaches, including richer, more immersive methods that engage affective and memory processes more deeply—such as secure base writing tasks, attachment figure visualization, and enhanced guided imagery. Comparative research examining multiple priming methods would help identify the most effective approaches for activating and deactivating the attachment system in experimental settings.

Another methodological limitation involves the absence of a formal suspicion probe or funnel debrief to assess whether participants inferred the purpose of the study or perceived a connection between the priming task and the lexical decision task. Given the elaborative supraliminal nature of the guided-imagery procedure, participants may theoretically form hypotheses about the study aims. Although no participant spontaneously expressed such awareness during the standardized debriefing, future research should incorporate structured awareness checks to better document potential demand characteristics and establish exclusion criteria based on participant awareness.

The conscious processing of attachment-related stimuli in this study may engage different neural pathways and brain regions linked to threat (e.g., amygdala and insula) and safety processing (e.g., ventral striatum, mPFC; Vrticka et al., 2008) compared to previously used subliminal methods. This processing likely exhibits different temporal dynamics and may not reflect how attachment system activation operates in naturalistic social contexts, where activation persists longer and receives continuous environmental reinforcement.

The lexical decision task (LDT), while valuable, has inherent limitations. It measures recognition speed rather than deeper, nuanced semantic processing, potentially missing subtler activation patterns relevant to attachment schemas. Individual differences in reading fluency, attention, and general cognitive speed can influence performance, potentially confounding the interpretation. The artificial nature of the task limits ecological validity, as it does not replicate naturalistic processing of relational cues. Because LDT captures only the earliest stages of word processing, future research should incorporate electrophysiological techniques that can track cognitive activity across the full time course. Event-related potentials (ERPs), which offer millisecond-level temporal resolution, would allow researchers to pinpoint the precise latency at which attachment primes begin to influence lexical accessibility—potentially revealing effects of attachment security that emerge in later processing stages beyond the window tapped by behavioral measures.

A further methodological limitation concerns the lexical composition of the stimulus set: all target words were nouns. This decision aligned with established attachment-priming paradigms using lexical decision tasks (e.g., Carr and Landau, 2012; Mikulincer et al., 2000) and reflected theoretical assumptions that internal working models primarily involve noun-like representations of attachment figures, relational states, and emotion-related themes (Baldwin and Meunier, 1999). Nouns also offered clearer psycholinguistic control in the present paradigm, as their frequency distributions, concreteness, and imageability ratings tend to be more stable and easier to match across categories than is typically feasible for verbs or adjectives. Moreover, psycholinguistic and neurocognitive research shows that verbs and adjectives impose additional processing demands—such as argument-structure integration, thematic-role assignment, or property attribution—and may engage partially distinct neural pathways during lexical access (Kemmerer, 2015; Tyler et al., 2001; Vigliocco et al., 2011). These differences raise the possibility that attachment priming effects might vary across grammatical classes, either because of differences in morphosyntactic complexity or because internal working models privilege entity-based (noun-like) representations over action- or property-based ones. Consequently, restricting the stimulus set to nouns constrains the generalizability of the present findings. Future research should therefore examine whether attachment priming effects extend to carefully matched sets of verbs and adjectives, and apply neurocognitive methods (e.g., ERP or fMRI) to determine the processing stages at which such divergences may emerge.

The laboratory setting with individual testing sessions may not reflect how attachment priming operates in naturalistic social contexts or group settings. Future studies should employ more ecological, longer-term manipulations or naturalistic observations to examine how attachment priming affects actual social behavior and decision-making in relational contexts. Additionally, physiological indicators of attachment activation (e.g., cortisol and sympathetic activity) alongside behavioral and self-report measures would enhance understanding of how different processing levels respond to supraliminal versus subliminal attachment cues.

The finding that individuals with low attachment anxiety show a strong relative response to security priming compared with the non-primed reference condition raises important questions about optimizing security-enhancing interventions for those with high attachment anxiety.

### 5.7 Constraints on generality (COG)

Following the recommendations of Simons et al. (2017) for transparent reporting of boundary conditions, I note that the generality of the present findings is limited by the specific characteristics of the sample, materials, and procedures. Importantly, a key theoretical boundary condition concerns the *supraliminal* nature of the attachment priming procedure. The guided-imagery task explicitly engaged conscious processing, allowing for elaboration of attachment-related representations and the recruitment of regulatory strategies shaped by chronic attachment orientations. Consequently, the observed effects—particularly the conditional effects of security priming as a function of attachment anxiety—reflect attachment system dynamics operating at a reflective, consciously accessible level. These effects may therefore not generalize to *subliminal priming or other fully automatic priming paradigms*, which are thought to engage less elaborative and more automatic attachment processes.

An additional constraint on generality concerns the lexical materials used to operationalize the semantic categories of interest. Each word category comprised only five items, which necessarily limits the breadth of semantic generalization that can be drawn from the present findings. Although supplementary linear mixed-effects models were employed to account for item-level variance, the estimation of random item effects is based on a relatively small stimulus pool. Accordingly, the observed effects should be interpreted as reflecting processing differences for the specific lexical instantiations included in this task, rather than for proximity- or distance-related semantic categories more broadly.

The study was conducted with young adult, predominantly female, Polish-speaking university students tested individually in laboratory settings. Consequently, the results should not be assumed to generalize to older adults, adolescents, clinical populations, or culturally and linguistically diverse groups. The use of a Polish lexical decision task and supraliminal guided-imagery priming represent specific methodological choices; effects may differ when other priming modalities, languages, or tasks involving deeper semantic or interpersonal processing are used. Furthermore, moderation patterns—particularly those involving attachment anxiety—require replication in larger and more heterogeneous samples. Thus, the present conclusions are expected to generalize primarily to populations and contexts closely resembling those examined in this study.

## 6 Conclusion

This study advances attachment theory by demonstrating that attachment security is not an automatic cognitive default, but a conditionally regulated state whose cognitive accessibility depends on individuals’ attachment-related regulatory strategies. In the context of supraliminal, consciously elaborated attachment primes, relative differences in proximity-related reaction times between the security priming condition and the non-primed reference condition emerged only at low levels of attachment anxiety, whereas no such differences were observed at higher levels of anxiety.

Supraliminal attachment priming via guided imagery yielded significant condition-related differences for attachment-related mental representations, but not for non-attachment concepts, supporting the domain specificity of attachment processes. This pattern was corroborated not only by condition effects for proximity- and distance-related words, but also by Bayesian analyses providing affirmative evidence for the absence of priming effects in positive, negative, and neutral word categories. Participants in the insecurity priming condition showed consistently faster reaction times to both proximity- and distance-related words compared to the non-primed reference condition, consistent with the attachment system’s dual mandate to seek closeness while retaining readiness for withdrawal under threat. Reaction times to distance-related words did not differ between the security priming and non-primed reference conditions, and attachment avoidance did not moderate these effects.

Together, these findings indicate that supraliminal security and insecurity primes are associated with distinct patterns of relative between-condition differences in attachment-related cognitive accessibility, with insecurity priming showing broadly consistent differences relative to the non-primed reference condition, and security-related differences emerging only under conditions of regulatory congruence. More broadly, the results underscore the conditional—rather than automatic—nature of attachment security when attachment cues are processed at a consciously elaborated level.

Taken together, these findings highlight the importance of considering level of awareness and regulatory context when interpreting the cognitive consequences of attachment security activation. Future research should examine whether similar conditional patterns emerge in clinical populations, across different cultural contexts, and when alternative priming or measurement approaches are used. From an applied perspective, the domain-specific and conditionally regulated nature of attachment priming effects suggests that interventions targeting attachment-related cognitions may be most effective when tailored to individuals’ regulatory strategies and levels of attachment anxiety.

## Data Availability

The datasets presented in this study can be found in online repositories. The names of the repository/repositories and accession number(s) can be found: all data and materials are publicly available on the Open Science Framework: https://doi.org/10.17605/OSF.IO/Y3J28.
