# Supplementary material for: The cognitive compass of attachment: how primed security and insecurity navigate mental representations
Source: Front Psychol. 2026 Feb 6;17:1713752. doi: 10.3389/fpsyg.2026.1713752 (PMC12920471; doi:10.3389/fpsyg.2026.1713752)
Supplement: Supplementary file 1 [file Table_1.docx]

| **Variable** | | **Attachment anxiety** | | **Attachment avoidance** | | **Felt security** | | **Trait anxiety** | | **Proximity-related words** | | **Distance-related words** | | **Positive words** | | **Negative words** | | **Neutral words** | |
| --- | --- | --- | --- | --- | --- | --- | --- | --- | --- | --- | --- | --- | --- | --- | --- | --- | --- | --- | --- |
| **Attachment anxiety** |  | — |  |  |  |  |  |  |  |  |  |  |  |  |  |  |  |  |  |
| **Attachment avoidance** |  | 0.38 | ** | — |  |  |  |  |  |  |  |  |  |  |  |  |  |  |  |
| **Felt security** |  | -0.14 |  | -0.15 |  | — |  |  |  |  |  |  |  |  |  |  |  |  |  |
| **Trait anxiety** |  | 0.66 | *** | 0.23 |  | -0.11 |  | — |  |  |  |  |  |  |  |  |  |  |  |
| **Proximity words** |  | -0.28 | * | -0.18 |  | 0.15 |  | -0.22 |  | — |  |  |  |  |  |  |  |  |  |
| **Distance words** |  | -0.18 |  | 0.02 |  | 0.24 | * | -0.20 |  | 0.66 | *** | — |  |  |  |  |  |  |  |
| **Positive words** |  | -0.05 |  | 0.14 |  | 0.07 |  | -0.11 |  | 0.56 | *** | 0.56 | *** | — |  |  |  |  |  |
| **Negative words** |  | -0.19 |  | 0.03 |  | 0.14 |  | -0.16 |  | 0.62 | *** | 0.58 | *** | 0.61 | *** | — |  |  |  |
| **Neutral words** |  | -0.14 |  | 0.03 |  | 0.03 |  | -0.18 |  | 0.74 | *** | 0.67 | *** | 0.69 | *** | 0.67 | *** | — |  |
| **Age** |  | 0.05 |  | 0.22 |  | -0.07 |  | 0.10 |  | 0.13 |  | 0.05 |  | 0.06 |  | 0.15 |  | 0.16 |  |
