# Supplementary material for: The cognitive compass of attachment: how primed security and insecurity navigate mental representations
Source: Front Psychol. 2026 Feb 6;17:1713752. doi: 10.3389/fpsyg.2026.1713752 (PMC12920471; doi:10.3389/fpsyg.2026.1713752)
Supplement: Supplementary file 2 [file Table_2.docx]

| **Variable** | | **Anxiety** | | **Avoidance** | | **Felt security** | | **Trait anxiety** | | **Proximity** | | **Distance** | | **Positive** | | **Negative** | | **Neutral** | |
| --- | --- | --- | --- | --- | --- | --- | --- | --- | --- | --- | --- | --- | --- | --- | --- | --- | --- | --- | --- |
| **Security priming  (*n* = 25)** |  |  |  |  |  |  |  |  |  |  |  |  |  |  |  |  |  |  |  |
| **Anxiety ^a^** |  | — |  |  |  |  |  |  |  |  |  |  |  |  |  |  |  |  |  |
| **Avoidance ^b^** |  | 0.54 | ** | — |  |  |  |  |  |  |  |  |  |  |  |  |  |  |  |
| **Felt security** |  | -0.20 |  | -0.51 | ** | — |  |  |  |  |  |  |  |  |  |  |  |  |  |
| **Trait anxiety** |  | 0.68 | *** | 0.42 | * | -0.11 |  | — |  |  |  |  |  |  |  |  |  |  |  |
| **Proximity words** |  | -0.03 |  | -0.32 |  | -0.18 |  | 0.08 |  | — |  |  |  |  |  |  |  |  |  |
| **Distance words** |  | -0.16 |  | -0.06 |  | -0.37 |  | -0.25 |  | 0.65 | *** | — |  |  |  |  |  |  |  |
| **Positive words** |  | -0.04 |  | -0.13 |  | -0.40 | * | -0.04 |  | 0.62 | *** | 0.70 | *** | — |  |  |  |  |  |
| **Negative words** |  | -0.24 |  | -0.27 |  | -0.14 |  | -0.04 |  | 0.61 | ** | 0.58 | ** | 0.57 | ** | — |  |  |  |
| **Neutral words** |  | -0.17 |  | -0.15 |  | -0.23 |  | -0.15 |  | 0.68 | *** | 0.76 | *** | 0.76 | *** | 0.55 | ** | — |  |
| **Age** |  | 0.03 |  | 0.19 |  | -0.14 |  | 0.20 |  | -0.16 |  | -0.17 |  | -0.17 |  | -0.26 |  | -0.01 |  |
| **Insecurity priming (*n* = 23)** |  |  |  |  |  |  |  |  |  |  |  |  |  |  |  |  |  |  |  |
| **Avoidance ^b^** |  | 0.44 | * | — |  |  |  |  |  |  |  |  |  |  |  |  |  |  |  |
| **Felt security** |  | -0.01 |  | 0.05 |  | — |  |  |  |  |  |  |  |  |  |  |  |  |  |
| **Trait anxiety** |  | 0.66 | *** | 0.18 |  | -0.02 |  | — |  |  |  |  |  |  |  |  |  |  |  |
| **Proximity words** |  | -0.42 | * | -0.30 |  | -0.11 |  | -0.60 | ** | — |  |  |  |  |  |  |  |  |  |
| **Distance words** |  | 0.04 |  | -0.18 |  | -0.30 |  | -0.33 |  | 0.59 | ** | — |  |  |  |  |  |  |  |
| **Positive words** |  | 0.14 |  | 0.35 |  | 0.05 |  | -0.13 |  | 0.34 |  | 0.31 |  | — |  |  |  |  |  |
| **Negative words** |  | 0.18 |  | 0.26 |  | 0.24 |  | -0.12 |  | 0.16 |  | 0.16 |  | 0.80 | *** | — |  |  |  |
| **Neutral words** |  | 0.23 |  | 0.11 |  | -0.03 |  | -0.11 |  | 0.49 | * | 0.53 | ** | 0.80 | *** | 0.67 | *** | — |  |
| **Age** |  | -0.13 |  | -0.05 |  | 0.23 |  | -0.14 |  | 0.10 |  | -0.26 |  | -0.14 |  | 0.03 |  | -0.15 |  |
| **Control condition  (*n* = 22)** |  |  |  |  |  |  |  |  |  |  |  |  |  |  |  |  |  |  |  |
| **Avoidance ^b^** |  | 0.12 |  | — |  |  |  |  |  |  |  |  |  |  |  |  |  |  |  |
| **Felt security** |  | -0.30 |  | 0.08 |  | — |  |  |  |  |  |  |  |  |  |  |  |  |  |
| **Trait anxiety** |  | 0.64 | ** | 0.05 |  | -0.29 |  | — |  |  |  |  |  |  |  |  |  |  |  |
| **Proximity words** |  | -0.45 | * | 0.02 |  | 0.09 |  | -0.18 |  | — |  |  |  |  |  |  |  |  |  |
| **Distance words** |  | -0.33 |  | 0.32 |  | 0.23 |  | -0.07 |  | 0.65 | ** | — |  |  |  |  |  |  |  |
| **Positive words** |  | -0.22 |  | 0.27 |  | -0.04 |  | -0.15 |  | 0.65 | *** | 0.51 | * | — |  |  |  |  |  |
| **Negative words** |  | -0.36 |  | 0.19 |  | -0.05 |  | -0.29 |  | 0.72 | *** | 0.64 | ** | 0.54 | ** | — |  |  |  |
| **Neutral words** |  | -0.36 |  | 0.19 |  | -0.02 |  | -0.22 |  | 0.85 | *** | 0.65 | *** | 0.57 | ** | 0.74 | *** | — |  |
| **Age** |  | 0.27 |  | 0.50 | * | -0.30 |  | 0.33 |  | 0.29 |  | 0.37 |  | 0.40 |  | 0.44 | * | 0.46 | * |
