# Supplementary material for: The cognitive compass of attachment: how primed security and insecurity navigate mental representations
Source: Front Psychol. 2026 Feb 6;17:1713752. doi: 10.3389/fpsyg.2026.1713752 (PMC12920471; doi:10.3389/fpsyg.2026.1713752)
Supplement: Supplementary file 3 [file Table_3.docx]

| **Dependent variable** | **Insecurity priming  (*n* = 23)** | **Security priming (*n* = 25)** | **Control condition  (*n* = 22)** |
| --- | --- | --- | --- |
| **Proximity words** | 729.95 (22.39) | 832.17 (29.23) | 887.52 (42.59) |
| **Distance words** | 777.79 (22.19) | 1025.00 (57.56) | 961.27 (53.11) |
| **Positive words** | 834.35 (50.51) | 958.09 (49.21) | 908.38 (57.57) |
| **Negative words** | 813.73 (27.87) | 914.90 (36.75) | 912.02 (55.87) |
| **Neutral words** | 869.37 (37.80) | 950.87 (47.93) | 1009.20 (58.30) |
