# Supplementary material for: The cognitive compass of attachment: how primed security and insecurity navigate mental representations
Source: Front Psychol. 2026 Feb 6;17:1713752. doi: 10.3389/fpsyg.2026.1713752 (PMC12920471; doi:10.3389/fpsyg.2026.1713752)
Supplement: Supplementary file 4 [file Table_4.docx]

| **Category** | **Model** | **P(M)** | **P(M\|data)** | **BF_M** | **BF₁₀** | **Error %** | **Effect** | **P(incl)** | **P(incl\|data)** | **BF Inclusion** |
| --- | --- | --- | --- | --- | --- | --- | --- | --- | --- | --- |
| **Proximity words** | Null model | 0.25 | 0.05 | 0.15 | 1.00 |  |  |  |  |  |
|  | Condition | 0.25 | 0.50 | 3.04 | 10.38 | 0.02 |  |  |  |  |
|  | Trait anxiety | 0.25 | 0.05 | 0.16 | 1.01 | 0.00 |  |  |  |  |
|  | Condition + Trait anxiety | 0.25 | 0.40 | 1.99 | 8.21 | 2.14 |  |  |  |  |
|  |  |  |  |  |  |  | Condition | 0.50 | 0.90 | 9.23 |
|  |  |  |  |  |  |  | Trait anxiety | 0.50 | 0.45 | 0.81 |
| **Distance words** | Null model | 0.25 | 0.03 | 0.10 | 1.00 |  |  |  |  |  |
|  | Condition | 0.25 | 0.89 | 25.49 | 27.19 | 0.01 |  |  |  |  |
|  | Trait anxiety | 0.25 | 0.01 | 0.02 | 0.18 | 0.00 |  |  |  |  |
|  | Condition + Trait anxiety | 0.25 | 0.07 | 0.21 | 2.02 | 1.34 |  |  |  |  |
|  |  |  |  |  |  |  | Condition | 0.50 | 0.96 | 24.82 |
|  |  |  |  |  |  |  | Trait anxiety | 0.50 | 0.07 | 0.08 |
| **Positive words** | Null model | 0.25 | 0.54 | 3.59 | 1.00 |  |  |  |  |  |
|  | Condition | 0.25 | 0.20 | 0.74 | 0.36 | 0.04 |  |  |  |  |
|  | Trait anxiety | 0.25 | 0.19 | 0.71 | 0.35 | 0.00 |  |  |  |  |
|  | Condition + Trait anxiety | 0.25 | 0.07 | 0.21 | 0.12 | 0.82 |  |  |  |  |
|  |  |  |  |  |  |  | Condition | 0.50 | 0.26 | 0.36 |
|  |  |  |  |  |  |  | Trait anxiety | 0.50 | 0.26 | 0.35 |
| **Negative words** | Null model | 0.25 | 0.43 | 2.27 | 1.00 |  |  |  |  |  |
|  | Condition | 0.25 | 0.23 | 0.88 | 0.52 | 0.02 |  |  |  |  |
|  | Trait anxiety | 0.25 | 0.23 | 0.90 | 0.54 | 0.00 |  |  |  |  |
|  | Condition + Trait anxiety | 0.25 | 0.11 | 0.38 | 0.26 | 2.56 |  |  |  |  |
|  |  |  |  |  |  |  | Condition | 0.50 | 0.34 | 0.51 |
|  |  |  |  |  |  |  | Trait anxiety | 0.50 | 0.34 | 0.52 |
| **Neutral words** | Null model | 0.25 | 0.40 | 2.04 | 1.00 |  |  |  |  |  |
|  | Trait anxiety | 0.25 | 1.02 | 0.62 |  | 0.00 |  |  |  |  |
|  | Condition | 0.25 | 0.22 | 0.86 | 0.55 | 0.02 |  |  |  |  |
|  | Condition + Trait anxiety | 0.25 | 0.12 | 0.40 | 0.29 | 1.23 |  |  |  |  |
|  |  |  |  |  |  |  | Trait anxiety | 0.50 | 0.37 | 0.59 |
|  |  |  |  |  |  |  | Condition | 0.50 | 0.34 | 0.52 |

*Note*. Table presents full Bayesian ANCOVA results, including model comparison and effect-inclusion Bayes factors. **P(M)** = prior model probability; **P(M|data)** = posterior model probability; **BF_M** = Bayes factor comparing each model to the full set of competing models; **BF₁₀** = Bayes factor comparing the model including Condition to the null model; **Error %** = numerical approximation error. **P(incl)** = prior inclusion probability; **P(incl|data)** = posterior inclusion probability; **BF Inclusion** = Bayes factor comparing matched models with versus without a given effect. Higher BF values indicate stronger evidence for the corresponding model or effect. The analysis was conducted on raw RTs to preserve interpretability of latency differences in ms.
