# Supplementary material for: The cognitive compass of attachment: how primed security and insecurity navigate mental representations
Source: Front Psychol. 2026 Feb 6;17:1713752. doi: 10.3389/fpsyg.2026.1713752 (PMC12920471; doi:10.3389/fpsyg.2026.1713752)
Supplement: Supplementary file 5 [file Table_5.docx]

| **Comparison** | ***n*1** | ***n*2** | ***d*** | ***df*** | **Hedges *g*** | **SE(g)** | **95% CI Lower** | **95% CI Upper** |
| --- | --- | --- | --- | --- | --- | --- | --- | --- |
| **Security vs. Control** | 25 | 22 | 1.65 | 45 | 1.62 | 0.34 | 0.96 | 2.29 |
| **Security vs. Insecurity** | 25 | 23 | -2.86 | 46 | -2.81 | 0.41 | -3.62 | -2.01 |
| **Control vs. Insecurity** | 22 | 23 | -1.22 | 43 | -1.2 | 0.33 | -1.84 | -0.56 |

*Note*. Effect sizes reflect Hedges’ g (bias-corrected standardized mean difference). Standard errors and confidence intervals were computed based on group sample sizes using the conventional formula for the standard error of standardized mean differences.
