# Supplementary material for: The cognitive compass of attachment: how primed security and insecurity navigate mental representations
Source: Front Psychol. 2026 Feb 6;17:1713752. doi: 10.3389/fpsyg.2026.1713752 (PMC12920471; doi:10.3389/fpsyg.2026.1713752)
Supplement: Supplementary file 6 [file Table_6.docx]

**S6_Linear mixed-effects model (LMM) for log-transformed reaction times in LDT**

Given that each semantic category in the lexical decision task contained only five items presented once, item-level idiosyncrasies may may obscure or limit the detectability of Condition × Category effects when analyses are based on aggregated reaction times. To evaluate whether the primary findings were robust to this hierarchical structure, I fitted a supplementary trial-level linear mixed-effects model (LMM) treating participants and items as crossed random intercepts, with random slopes for Category where identifiable. All analyses were conducted in R (version 2024.09.1) using the lme4, lmerTest, and emmeans packages.

Reaction times were log-transformed to reduce positive skewness and stabilize variance, a variance-stabilizing transformation that improves model assumptions but can reduce sensitivity to additive effects expressed in milliseconds. The fixed-effects structure included Condition, Word Category, and their interaction. Although the small number of items per category limits the sensitivity of mixed models to detect Condition × Category interactions, LMMs provide a conservative test in this specific design, where the limited number of items constrains the estimation of random effects.

The model revealed no significant main effect of Condition, *F*(2, 62.8) = 1.47, *p* = .232, no main effect of Category, *F*(4, 24.2) = 2.03, *p* = .125, and no Condition × Category interaction, *F*(8, 1396) = 0.14, *p* = .983. Bonferroni-adjusted pairwise contrasts were also nonsignificant. The absence of a main effect of Category in the LMM likely reflects reduced statistical power at the item level—given the small number of items per category—rather than the absence of substantive differences in lexical accessibility across semantic categories.Importantly, the LMM did not reveal effects in directions contradicting the aggregated RT analyses. Together with the log-transformed sensitivity analyses reported in S7.1, this model supports the robustness of the main findings across different analytic choices.

| **Random Effects** |  |  |  |  |  |
| --- | --- | --- | --- | --- | --- |
| **Effect** | **Variance** | ***SD*** |  |  |  |
| ID (Intercept) | 0.03267 | 0.18074 |  |  |  |
| item (Intercept) | 0.00409 | 0.06395 |  |  |  |
| Residual | 0.05518 | 0.23491 |  |  |  |
| **Fixed Effects** |  |  |  |  |  |
| **Predictor** | **Estimate** | ***SE*** | ***df*** | ***p*** |  |
| Intercept | 6.70 | 0.06 | 101 | < .001 |  |
| Condition: security | –0.09 | 0.07 | 99 | .198 |  |
| Condition: insecurity | 0.01 | 0.07 | 101 | .878 |  |
| Category: distance | 0.09 | 0.05 | 48 | .086 |  |
| Category: positive | 0.00 | 0.05 | 49 | .983 |  |
| Category: negative | 0.00 | 0.05 | 49 | .975 |  |
| Category: neutral | 0.08 | 0.05 | 48 | .126 |  |
| Security × Distance | 0.01 | 0.05 | 1396 | .811 |  |
| Insecurity × Distance | 0.00 | 0.05 | 1396 | .971 |  |
| Security × Positive | 0.04 | 0.05 | 1396 | .392 |  |
| Insecurity × Positive | 0.02 | 0.05 | 1396 | .637 |  |
| Security × Negative | 0.01 | 0.05 | 1396 | .758 |  |
| Insecurity × Negative | 0.04 | 0.05 | 1396 | .391 |  |
| Security × Neutral | 0.01 | 0.05 | 1396 | .764 |  |
| Insecurity × Neutral | 0.01 | 0.05 | 1396 | .810 |  |
| **Type III ANOVA Tests (Satterthwaite)** |  |  |  |  |  |
| **Effect** | **Sum Sq** | **Mean Sq** | ***Num df*** | ***Den df*** | ***p*** |
| Condition | 0.17 | 0.08 | 2 | 62.8 | .232 |
| Category | 0.44 | 0.11 | 4 | 24.2 | .125 |
| Condition × Category | 0.11 | 0.01 | 8 | 1396 | .983 |
| **Pairwise Contrasts (Bonferroni-adjusted)** |  |  |  |  |  |
| **Proximity** |  |  |  |  |  |
| **Contrast** | **Estimate** | ***SE*** | ***t*** | ***p*** |  |
| control – security | 0.09 | 0.07 | 1.27 | .618 |  |
| control – insecurity | –0.01 | 0.07 | –0.15 | 1.000 |  |
| security – insecurity | –0.10 | 0.06 | –1.47 | .434 |  |
| **Distance** |  |  |  |  |  |
| **Contrast** | **Estimate** | ***SE*** | ***t*** | ***p*** |  |
| control – security | 0.07 | 0.07 | 1.10 | .820 |  |
| control – insecurity | –0.01 | 0.07 | –0.18 | 1.000 |  |
| security – insecurity | –0.09 | 0.06 | –1.33 | .563 |  |
| **Positive** |  |  |  |  |  |
| **Contrast** | **Estimate** | ***SE*** | ***t*** | ***p*** |  |
| control – security | 0.04 | 0.07 | 0.66 | 1.000 |  |
| control – insecurity | –0.03 | 0.07 | –0.49 | 1.000 |  |
| security – insecurity | –0.08 | 0.06 | –1.19 | .707 |  |
| **Negative** |  |  |  |  |  |
| **Contrast** | **Estimate** | ***SE*** | ***t*** | ***p*** |  |
| control – security | 0.07 | 0.07 | 1.05 | .885 |  |
| control – insecurity | –0.05 | 0.07 | –0.77 | 1.000 |  |
| security – insecurity | –0.12 | 0.06 | –1.89 | .184 |  |
| **Neutral** |  |  |  |  |  |
| **Contrast** | **Estimate** | ***SE*** | ***t*** | ***p*** |  |
| control – security | 0.07 | 0.07 | 1.05 | .883 |  |
| control – insecurity | –0.02 | 0.07 | –0.32 | 1.000 |  |
| security – insecurity | –0.09 | 0.07 | –1.43 | .470 |  |

*Note.* Trial-level analyses included participants with available raw LDT logs (*n* = 63); the full sample
(*N* = 70) was retained in the aggregated analyses because summary RTs had been computed prior to the loss of several raw logs during routine data management. DV = log-transformed reaction times (RTs, in milliseconds) for correct lexical-decision trials. All parameter estimates correspond to effects on the log-RT scale. Fixed-effect coefficients (b) are estimates from the linear mixed-effects model logRT ~ Condition × Category + (1 | ID) + (1 | item), fitted via maximum likelihood. *SE* = standard error; df = Satterthwaite-approximated degrees of freedom; *p* = two-tailed. Reference levels were Condition = control and Category = proximity. Random intercepts were specified for participants and items. No Condition or Condition × Category effects reached significance; adjusted *p*-values of 1.000 reflect Bonferroni-corrected values truncated at the upper bound.
