# Supplementary material for: The cognitive compass of attachment: how primed security and insecurity navigate mental representations
Source: Front Psychol. 2026 Feb 6;17:1713752. doi: 10.3389/fpsyg.2026.1713752 (PMC12920471; doi:10.3389/fpsyg.2026.1713752)
Supplement: Supplementary file 7 [file Table_7.docx]

**S7_Robustness and Sensitivity Analyses**

**S7.1 Log-transformed RT analyses**

To evaluate the robustness of the primary findings with respect to skewness in the RT distributions, all multivariate and univariate analyses were repeated using log-transformed RTs for the five word categories. Trait anxiety was entered as a covariate, and Condition (security, insecurity, control) served as the between-subjects factor, mirroring the primary analyses. Log-transformation substantially improved normality and reduced heterogeneity of covariance matrices, and all substantive conclusions remained unchanged.

Multivariate tests for the log-transformed RTs indicated a significant multivariate effect of Condition (Table S7.1.1). Follow-up univariate ANCOVAs (Table S7.1.2) showed that Condition significantly predicted log-transformed RTs for proximity and distance words, but not for the remaining categories. Bonferroni-adjusted pairwise comparisons for these significant outcomes are reported in Table S7.1.3. The pattern of effects closely mirrored the analyses based on untransformed RTs, indicating that the main findings are robust to log-transformation.

## Table S7.1.1

## *Multivariate Tests for Log-Transformed RTs (MANOVA)*

| **Test** | **Value** | ***F*** | ***df* (hyp)** | ***df* (error)** | ***p*** | **Partial η²** |
| --- | --- | --- | --- | --- | --- | --- |
| **Pillai’s Trace** | 0.34 | 2.57 | 10 | 126 | .007 | .17 |
| **Wilks’ Lambda** | 0.69 | 2.55 | 10 | 124 | .008 | .17 |
| **Hotelling’s Trace** | 0.42 | 2.54 | 10 | 122 | .008 | .17 |
| **Roy’s Largest Root** | 0.28 | 3.47 | 5 | 63 | .008 | .22 |

## Table S7.1.2

## *Univariate ANCOVA Results for Log-Transformed RTs*

| **Dependent Variable** | ***F*(2,66)** | ***p*** | **Partial η²** |
| --- | --- | --- | --- |
| **logProximity** | 5.20 | .008 | .136 |
| **logDistance** | 6.67 | .002 | .168 |
| **logPositive** | 1.67 | .196 | .048 |
| **logNegative** | 1.54 | .223 | .045 |
| **logNeutral** | 1.50 | .231 | .043 |

*Note.* Dependent Variable - log-transformed RT variables.

## Table S7.1.3

## *Pairwise Comparisons for Significant Outcomes (Bonferroni-adjusted)*

| **Comparison** | **Mean Difference** | ***SE*** | ***p* (adj.)** | **95% CI** |
| --- | --- | --- | --- | --- |
| **DV: logProximity** | | | | |
| **Insecurity – Control** | -0.17 | 0.06 | .008 | [-0.305, -0.036] |
| **Insecurity – Security** | -0.12 | 0.05 | .071 | [-0.252, 0.007] |
| **Security – Control** | -0.05 | 0.05 | 1.000 | [-0.179, 0.084] |
| **DV: logDistance** | | | | |
| **Insecurity – Security** | -0.24 | 0.07 | .002 | [-0.410, -0.076] |
| **Insecurity – Control** | -0.17 | 0.07 | .051 | [-0.346, 0.000] |
| **Security – Control** | 0.07 | 0.07 | .935 | [-0.099, 0.239] |

*Note*. DV = log-transformed RT variables. Trait anxiety included as covariate. Values reflect multivariate tests for Condition*. F*-tests assess between-group differences in estimated marginal means of log-transformed RTs after controlling for trait anxiety. Pairwise comparisons are Bonferroni-adjusted. Negative mean differences indicate faster responses in the first-listed condition. Full SPSS outputs for MANOVA, ANCOVAs, and pairwise contrasts (log-transformed RTs) are provided in the OSF repository at: <https://osf.io/y3j28/overview?view_only=24d5f4eabc9e49d4986318392d8c2771>

**S7.2 Accuracy and a brief assessment of speed–accuracy trade-offs**

Trial-level accuracy was analysed for the subset of participants for whom complete LDT log files were available (*n* = 63; 1,534 word trials after trimming RTs < 250 ms and > 2,000 ms). Accuracy for each word trial was coded as correct vs. incorrect (corr = 1/0). Words were assigned to five semantic categories (proximity, distance, positive, negative, neutral) using the PEBL category code or, when unavailable, the predefined mapping used in the main RT analyses. Priming Condition (security, insecurity, control) was entered as a between-subjects factor, Category as a within-item factor, and participants and items were treated as crossed random effects. Accuracy by Condition × Category is summarised in Table S7.2.

Accuracy was uniformly high across experimental cells. Mean accuracy ranged from .91 to 1.00 across Condition × Category combinations, with most cell means exceeding .95 (Table S7.2). Because error trials were sparse in several cells, the mixed-effects logistic model
**corr ~ Condition × Category + (1 | ID) + (1 | item)** exhibited signs of quasi-separation and a degenerate Hessian. Consistent with the descriptive pattern, none of the fixed effects reached significance (all *p*s > .22), indicating that neither Condition nor the Condition × Category interaction accounted for meaningful variance in accuracy.

To examine whether the RT effects could reflect a speed–accuracy trade-off, each participant’s mean RT (restricted to correct trials) was correlated with their mean accuracy across all word categories. Mean RT and mean accuracy were essentially uncorrelated,
***r*(61) = –.02, 95% CI [–.27, .23], *p* = .88**, providing no evidence that faster responders were less accurate. Full R code along with the outputs are provided in the OSF repository at: <https://osf.io/y3j28/overview?view_only=24d5f4eabc9e49d4986318392d8c2771>

# **Table S7.2**

***Mean Lexical Decision Accuracy (Proportion Correct) by Priming Condition and Word Category***

| **Condition** | **Category** | ***M* Accuracy** | ***SD* Accuracy** | ***n*** | **95% CI** |
| --- | --- | --- | --- | --- | --- |
| **Control** | Proximity | 1.00 | 0.00 | 19 | [1.00, 1.00] |
|  | Distance | 0.99 | 0.05 | 19 | [0.97, 1.01] |
|  | Positive | 0.98 | 0.06 | 19 | [0.95, 1.01] |
|  | Negative | 0.97 | 0.10 | 19 | [0.92, 1.02] |
|  | Neutral | 0.99 | 0.05 | 19 | [0.97, 1.01] |
| **Security** | Proximity | 0.98 | 0.06 | 22 | [0.95, 1.00] |
|  | Distance | 0.96 | 0.08 | 22 | [0.93, 1.00] |
|  | Positive | 0.97 | 0.07 | 22 | [0.94, 1.00] |
|  | Negative | 0.99 | 0.04 | 22 | [0.98, 1.01] |
|  | Neutral | 0.96 | 0.08 | 22 | [0.93, 1.00] |
| **Insecurity** | Proximity | 0.91 | 0.20 | 22 | [0.82, 1.00] |
|  | Distance | 0.98 | 0.06 | 22 | [0.96, 1.01] |
|  | Positive | 0.97 | 0.10 | 22 | [0.92, 1.02] |
|  | Negative | 0.95 | 0.13 | 22 | [0.89, 1.01] |
|  | Neutral | 0.92 | 0.10 | 22 | [0.87, 0.98] |

*Note.* 95% CIs computed using normal approximation: M ± 1.96 × (SD / √N). Accuracy approaches ceiling in most conditions, yielding narrow intervals.

**S7.3 Permutation-based MANOVA sensitivity check - control of false positives**

To address concerns about multiple testing across the five RT outcomes and to provide a conservative, distribution-free sensitivity check of the omnibus multivariate Condition effect, I conducted a non-parametric permutation MANOVA using the subset of participants with complete trial-level LDT logs (n = 63). For each participant, log-transformed mean RTs were computed for all five semantic categories, and the multivariate model Y ~ Condition + TraitAnxiety was re-estimated across 10,000 random permutations of Condition labels. Pillai’s Trace was used as the multivariate test statistic. A summary of the permutation results is presented in Table S7.3, and the permutation distribution is shown in Figure S7.4. Complete R code and outputs are available in the OSF repository: <https://osf.io/y3j28/overview?view_only=24d5f4eabc9e49d4986318392d8c2771>

The observed Pillai’s Trace for Condition was V = 0.10, corresponding to the parametric F(10, 112) = 0.59, p = .82. The permutation-derived p-value was p_perm = .82, indicating no evidence for an omnibus multivariate Condition effect under permutation resampling. Given the conservative nature of an omnibus permutation test in a small sample with multiple outcomes, this result is interpreted as indicating that any Condition-related differences are not expressed as a strong global multivariate shift across all five RT categories. Instead, the primary effects observed in the main analyses are best characterized as category-specific, consistent with the domain-focused pattern reported for proximity and distance.

# **Table S7.3**

***Permutation-Based MANOVA for Log-Transformed Reaction Times (RTs)***

| **Effect** | **Pillai’s Trace (Observed)** | **Parametric *F*** | ***df(h), df(e)*** | **Parametric *p*** | **Permutation *p* (10,000 perms)** | **N** |
| --- | --- | --- | --- | --- | --- | --- |
| **Condition** | 0.10 | 0.59 | 10, 112 | .82 | **.82** | 63 |

Note. Permutation MANOVA was conducted using 10,000 random shuffles of Condition labels. Dependent variables were log-transformed mean RTs for proximity, distance, positive, negative, and neutral word categories. Trait anxiety was included as a covariate in both parametric and permutation models. The permutation p-value represents the proportion of permuted Pillai’s Trace values greater than or equal to the observed test statistic. Results indicate no evidence of a multivariate effect of Condition under permutation resampling. See also Figure S7.4 for the permutation distribution of Pillai’s Trace.

Together with the log-transformed MANOVA/ANCOVA results (Section S7.1) and the trial-level robustness analyses (S6 Table), these supplementary checks **support** the conclusion that the primary RT findings do not hinge on the raw RT scale and are not readily attributable to unmodelled item-level variability or speed–accuracy trade-offs, while the permutation MANOVA provides a conservative omnibus assessment relevant to familywise error control across outcomes.

**Figure S7.4**

*Permutation Distribution of Pillai’s Trace for the Multivariate Condition Effect on Log-Transformed RTs.*


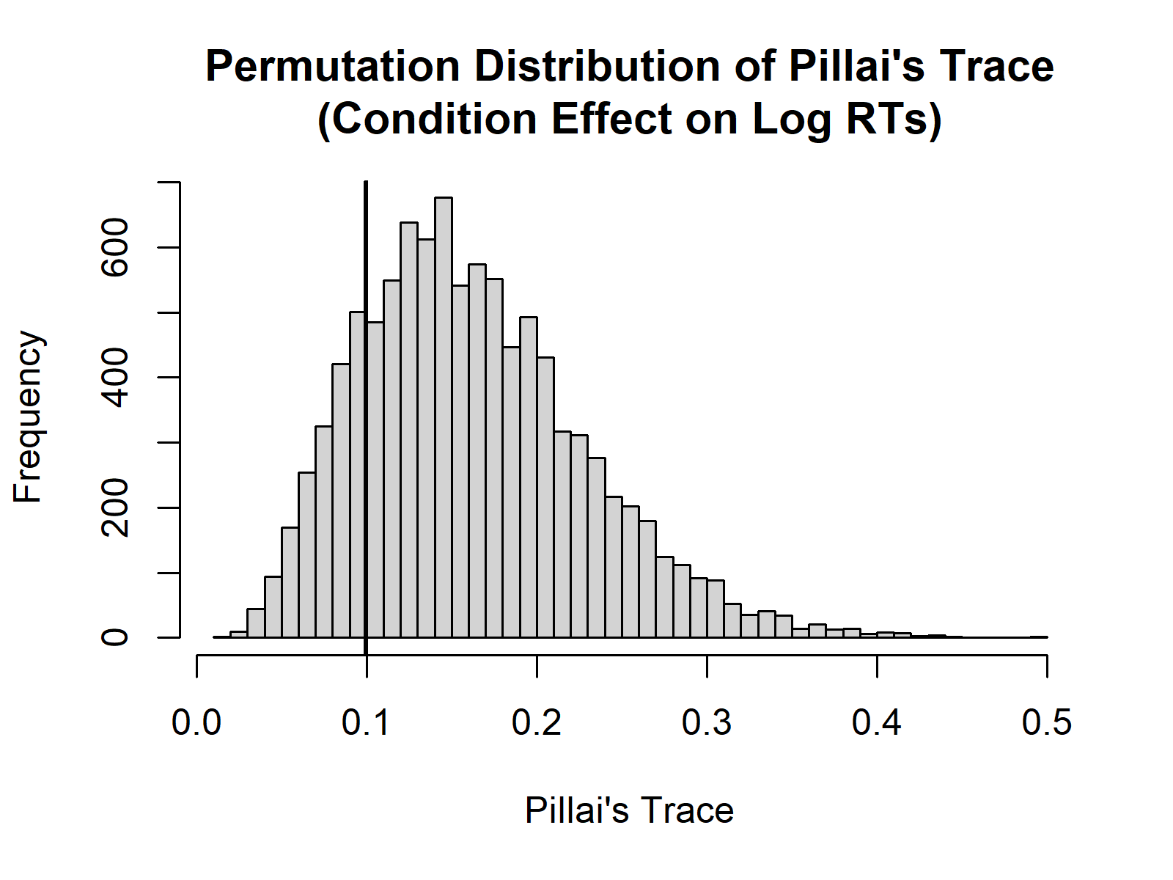


*Note.* The histogram shows the permutation distribution of Pillai’s Trace for the multivariate Condition effect on log-transformed mean RTs across the five semantic categories (proximity, distance, positive, negative, neutral) in the subset of participants with complete trial-level LDT logs (n = 63). For each of 10,000 permutations, Condition labels were randomly reassigned across participants while Trait Anxiety was retained as a covariate. The vertical line marks the observed Pillai’s Trace (*V* = 0.10), corresponding to the parametric *F*(10,112) = 0.59, p = .82. The permutation-derived *p*-value (*p*_perm = .82) indicates no evidence for a multivariate Condition effect under permutation resampling.
